# Single-cell and spatial transcriptomics reveal correlation between RNA methylation-related miRNA risk model and immune infiltration in hepatocellular carcinoma

**DOI:** 10.3389/fonc.2025.1553239

**Published:** 2025-05-09

**Authors:** Rong Su, Yong Du, Pan Tian, Weifang Ma, Yongfeng Hui, Shaoqi Yang

**Affiliations:** ^1^ Department of Gastroenterology, General Hospital of Ningxia Medical University, Yinchuan, Ningxia, China; ^2^ Department of Anesthesiology, People’s Hospital of Ningxia Hui Autonomous Region, Yinchuan, Ningxia, China; ^3^ Department of Neurology, General Hospital of Ningxia Medical University, Yinchuan, Ningxia, China; ^4^ Department of Hepatobiliary Surgery, General Hospital of Ningxia Medical University, Yinchuan, Ningxia, China

**Keywords:** RNA methylation, miRNAs, risk model, prognosis, immune microenvironment, single-cell RNA sequencing, spatial transcriptomics, hepatocellular carcinoma

## Abstract

**Introduction:**

Increasing evidence highlights the pivotal role of RNA methylation and miRNAs in hepatocellular carcinoma (HCC). However, the risk associated with RNA methylation-related miRNAs (RMRMs) in the HCC immune microenvironment remains largely unknown. Here, we predicted the correlation between RMRM risk and immune cell infiltration in HCC using machine learning.

**Methods:**

MiRNA sequencing data was used to identify RMRMs. A risk score model of HCC was developed utilizing four RMRMs, including miR-551a, miR-4739, miR-326, and miR-210-3p.

**Results:**

Patients with high-risk scores exhibited poorer prognoses. Single-cell RNA sequencing (scRNA-seq) analysis revealed the high-risk group exhibited increased infiltration levels of several immune cell subtypes, including myeloid-derived suppressor cell (MDSC), macrophage, and T cells. The data integration of scRNA-seq and bulk RNA-seq showed the decreased TIDE score in the high-risk patients and the elevated levels of Macro-secreted phosphoprotein 1 (SPP1), MDSC-meiotic nuclear divisions 1 (MND1), γδ T cells, and Macro-complement C1q C chain (C1QC) predicted adverse prognosis. ScRNA-seq and spatial transcriptomics data integration unveiled the spatial distribution of RMRMs risk scores and their correlation with immune cell subtype localization. Risk model-based clustering of HCC samples revealed that cluster 2, characterized by a higher risk score, correlated with a poorer prognosis and reduced immune and stromal scores. In vitro, the overexpression of miR-4739 in Huh-7 cells significantly induced SPP1^+^ macrophages, and the culture medium derived from SPP1^+^ macrophages further promoted the proliferation and migration of Huh-7 cells. Furthermore, miR-4739 reduced m1A methylation by inhibiting tRNA methyltransferase 61A (TRMT61A) expression.

**Discussion:**

Our study reveals that the RMRM risk model could effectively predict the prognosis of HCC, and SPP1^+^ macrophages regulated by miR-4739-RNA methylation promote the proliferation and migration of HCC cells. These results highlight the potential of RMRMs in predicting the prognosis of HCC.

## 1 Introduction

Hepatocellular carcinoma (HCC) is a highly aggressive form of cancer that imposes a significant burden on patients across the globe (1, 2). HCC is characterized by strong invasiveness, uncomplicated metastasis, and poor prognosis (3–5). Due to the lack of noticeable symptoms in the early stages of HCC, patients are often diagnosed when the disease has already progressed to an advanced stage (5). Current management options for HCC include traditional drug treatment, Radio-Frequency Ablation, systemic molecular, resections in regional, and liver transplants (6–8). However, these approaches are limited in their efficacy of HCC. The immune microenvironment plays a pivotal role in the development and progression of HCC, and immune checkpoint inhibitors have revolutionized the management of HCC (7, 9, 10). Although immunotherapy has achieved significant breakthroughs in the treatment of HCC, the clinical outcomes of HCC for advanced stages are still unfavorable due to the heterogeneity of HCC in terms of molecular and cellular signatures. Consequently, there is an urgent need to identify easily quantifiable immune-related biomarkers for early detection or therapeutic indicators of HCC.

MicroRNAs (miRNAs), known for their role in regulating gene expression, hold substantial promise in the prognostic evaluation and therapeutic management of HCC (11–17). In fact, the role of miRNAs in the immune response of HCC has been validated. For example, endoplasmic reticulum stress induces HCC cells to release exosomal miRNA-23a-3p, which in turn upregulates the expression of programmed death ligand 1 (PD-L1) in macrophages (18). MiRNA-223 can attenuate hepatocarcinogenesis by blocking hypoxia-driven angiogenesis and immunosuppression (19). Hu et al. indicated that miRNA-22 reduced the abundance of interleukin 17 (IL17)-producing T cells and inhibited IL17 signaling in HCC (20). These studies highlight the role and involvement of miRNAs in the immunity of HCC. Therefore, it is necessary to further explore the potential of miRNA as a biomarker for HCC and the underlying regulatory mechanisms of miRNA on the HCC immune microenvironment.

In recent years, up to 170 distinct chemical modifications have been identified in RNA molecules, with N1-methyladenosine (m1A), N6-methyladenosine (m6A), 5-methylcytosine (m5C), and N7-methylguanosine (m7G) being particularly significant (21). The role of RNA methylation has been revealed in HCC. Specifically, the m6A methyltransferase-like 3 (METTL3)promotes non-alcoholic fatty liver disease-induced HCC (22). The m1A methylation levels in tRNA are remarkably elevated in tumor tissues of HCC patients and are negatively associated with the survival of HCC patients (23). m5C is highly conserved in different species, and the distribution pattern of m5C in diversified RNA forms is species-specific (24). Tumor microenvironment (TME) and prognosis of HCC are significantly influenced by the patterns of m5C modification (25). Notably, a lower patient survival rate was significantly related to higher expression of almost all m5C regulators in HCC. The upregulation of tRNA m7G methyltransferase complex components METTL1 and WD repeat domain 4 has enhanced lapatinib resistance in HCC and increased sensitivity to METTL1-targeting therapies (26). MiRNAs are virtually involved at the post-transcriptional level and bind to 3’ UTR of their target mRNA to regulate gene expression (27). Some studies have indicated that miRNAs can bind RNA methylation-related genes to regulate HCC biological processes (28, 29). However, it is still unclear whether miRNAs regulate the HCC immune microenvironment by binding to RNA methylation-related genes.

To explore the relationships of m6A/m5C/m1A/m7G-related miRNAs with the immune microenvironment of HCC, we established a risk model of RNA methylation-related miRNAs (RMRMs) in HCC using micro RNA sequencing (miRNA-seq) data and machine learning algorithms. We verified their reliability in single-cell RNA sequencing (scRNA-seq) and spatial transcriptomic data. In this study, we identified four RMRMs associated with the risk model of HCC and assessed the effects of RNA methylation-related miR-4739 in macrophages on HCC cells. Our findings provide a new scientific basis for developing biomarkers and targeted therapeutic molecules for HCC based on immunology.

## 2 Materials and methods

### 2.1 MiRNA -seq data analysis

We downloaded miRNA-seq data in TCGA-LIHC samples from the Genomic Data Commons (GDC) database (https://portal.gdc.cancer.gov/). The DESeq2 package and Wilcoxon rank-sum test in R software were applied to analyze the differentially expressed miRNAs between HCC and normal tissues, with a |log2 Fold Change| > 1 criterion and adjust *P* value < 0.05. Based on previous studies (30, 31), M6A/m5C/m1A/m7G-associated genes were selected. Miranda and TargetScan tools were used to predict miRNA binding sites on the 3’ UTR of genes. The binding sites with Context+ Score > 0, Structure Score > 155, and Free Energy < -20 were selected. The regulatory network between miRNAs and RNA methylation-related genes was conducted utilizing Cytoscape (version 3.9.1).

### 2.2 HCC risk model construction based on RMRMs

The clinical characteristics of 372 TCGA-LIHC patients were downloaded from the cBioPortalData package in R software. The univariate Cox analysis was used to identify 28 RMRMs associated with the prognosis of patients with HCC (*P* value < 0.05). Lasso regression analysis was performed using the glmnet package in R software. Specifically, cancer samples in TCGA-LIHC were divided into training and testing sets, each containing 186 samples. The clinical information of both sets is presented in **Supplementary Tables S1** and **S2**. After performing Lasso regression analysis on the relationship between 28 RMRMs and prognosis, 13 RMRMs were obtained. Then, multivariate Cox analysis was performed based on the expression and survival of these 13 RMRMs, and 4 RMRMs had a *P* value less than 0.05.

For each sample, we calculated the risk score utilizing the following formula: Risk score = $exp^{\sum_{i=1}^{n}Coef_{i}\times Expr_{i}}$ . The survival ROC and ggplot2 packages were used to calculate and draw the ROC curves. Samples of low- and high-risk groups were generated following the median risk score. The optimal threshold was used to divide the samples of training/testing groups into high and low groups. Kaplan-Meier (KM) curves of the two groups were drawn utilizing the survival and survminer packages. The log-rank test compares survival differences between the two groups.

### 2.3 ScRNA-seq data analysis

The GSE202642 dataset was downloaded from the Gene Expression Omnibus (GEO) database, including 6 hepatitis B virus (HBV)-infected HCC samples and 4 HBV samples (32). The uniform manifold approximation and projection (UMAP) algorithm performed an overall dimensionality reduction analysis. The singleR package and BlueprintEncodeData were used as reference data for auxiliary annotation, followed by the FindAllMarkers database and previous studies to find marker genes for manual annotation of different clusters. The risk model derived from scRNA-seq data (GSE202642) was calculated using GSVA methodology based on 18 target genes of 4 miRNAs. According to the risk model, gene set variation analysis (GSVA) scores and immune cell infiltrations were analyzed for scRNA-seq data.

### 2.4 Spatial transcriptomics data analysis

Sequencing data was processed using the Space Ranger software (10× Genomics) for the demultiplexing process, transforming barcode and read data into FASTQ format, aligned with stained tissue imagery, and creating read count matrices. Subsequently, the processed data were analyzed via Seurat (v 4.0). The initial visualization of count data overlaid on tissue images to distinguish between technical and histological variances, subsequent removal of areas indicative of necrosis and tissue folding, and data normalization employing SCTransform. Post-filtering datasets were consolidated, and Harmony applied batch effect corrections across samples. Dimensionality reduction was achieved through PCA, and clustering was proceeded with the Leiden algorithm. The assignment of cell types to clusters was determined by analyzing the most variable genetic features.

### 2.5 Consensus clustering analysis

The Consensus Cluster Plus package was used to cluster HCC samples based on the expressions of 4 RMRMs with prognostic significance. The classification method selected k=2, meaning the HCC samples were divided into two groups, cluster 1 and cluster 2. Their KM curves and risk scores were analyzed. The CIBERSORT algorithm was used to evaluate the differences in immune cell infiltrations between cluster 1 and cluster 2. The Stromal, Immune, and ESTIMATE Score of TCGA samples were acquired from the following website: https://bioinformatics.mdanderson.org/public-software/estimate/.

### 2.6 Differential analysis

RNA-seq data in TCGA-LIHC was downloaded from GDC. The Wilcoxon rank-sum test in the R package was used to analyze the differentially expressed genes (DEGs, |log2 Fold Change| > 1, and adjust *P* value < 0.05) between HCC and paracancerous samples. We utilized the R package clusterProfiler to conduct GO and KEGG enrichment analyses.

### 2.7 Single nucleotide variation analysis

SNV data of TCGA-LIHC patients was downloaded from GDC. Maftools were used to plot waterfall charts of samples in cluster 1 and cluster 2.

### 2.8 Tumor mutation burden analysis

TMB data of TCGA-LIHC patients was downloaded from the cBioPortalData package in R software. The TMB was calculated based on tumor-specific gene mutations. We combined patient survival information and TMB data, divided all HCC samples into cluster 1 and cluster 2, and analyzed the survival status of HCC patients in cluster 1 and cluster 2. HCC patients were further divided into 4 clusters: low TMB + cluster 1, low TMB + cluster 2, high TMB + cluster 1, and high TMB + cluster 2. The KM curves of 4 clusters were depicted.

### 2.9 Drug sensitivity

The IC50 data for various drugs from liver cancer samples in TCGA was downloaded from a previous study (33). The IC_50_ between cluster 1 and cluster 2 was compared by t-test.

### 2.10 Clinical HCC tissues

The HCC tissue samples were obtained from the General Hospital of Ningxia Medical University. The patients signed an informed consent form before undergoing surgical resection. A total of 6 paracancerous tissue samples and 6 HCC tissue samples were obtained in the present study. The ethics committee of the General Hospital of Ningxia Medical University approved this study [KYLL-2022-1057].

### 2.11 qRT-PCR analysis

We extracted total RNA using TRIzol reagent (Invitrogen, CA, USA) according to the manufacturer’s protocol. After the RNA quality check, we reverse-transcribed RNA to cDNA. Random primer (1 μL) was added to a test tube, and the reaction was mixed with total RNA (1 μg) and RNase-Free ddH20 (added to 12 μL) for 5 min at 65°C. RT primer (1 μL), total RNA (1 μg), and RNase-Free ddH2O (add to 12 μL) were mixed and reacted at 65°C for 5 min. The resulting mixture (12 μL) was then mixed with 5×buffer (4 μL), dNTP Mix (2 μL), protector RNase inhibitor (1 μL), and transcriptase (1 μL), and the reaction was performed at 42°C for 60 min and at 70°C for 5 min. The resulting cDNA was stored at low temperatures. Kits used for qRT-PCR: 2 × Master Mix kit (Roche), reverse transcription kit (Thermo). A qRT-PCR instrument (ABI Q6, Applied Biosystems Inc., USA) was used for this experiment. We set the qRT-PCR parameters to 95°C for 10 min, 95°C for 15 s, and 60°C for 60 s × 45 amplification cycles. We standardized the expression levels to U6 and quantified the expression levels according to the 2^-ΔΔCT^ method. The primers of miR-4739, miR-210-3p, glyceraldehyde-3-phosphate dehydrogenase (GAPDH), and tRNA methyltransferase 61A (TRMT61A) used in this study are listed in **Table 1**.

**Table 1:** Primer sequences.

Gene	Primer sequences (5’–3’)
miR-4739-RT	GTCGTATCCAGTGCGTGTCGTGGAGTCGGCAAT TGCACTGGATACGACAGGGCCC
miR-210-3p-RT	GTCGTATCCAGTGCGTGTCGTGGAGTCGGCAAT TGCACTGGATACGACTCAGCCG
U6-F	CGATACAGAGAAGATTAGCATGGC
U6-R	AACGCTTCACGAATTTGCGT
miR-4739-F	GCAGAAGGGAGGAGGAG
miR-210-3p-F	GCTGTGCGTGTGACA
All-R	AGTGCGTGTCGTGGAGTCG

miR-XXX-RT: The primers are used for reverse transcription of miR-4739 and miR-210-3p. miR-4739-F and miR-210-3p-F: The forward primers are used for RT-qPCR analysis of miR-4739 and miR-210-3p. All-R: The reverse primers are used for qRT-PCR analysis of miR-4739 and miR-210-3p.

### 2.12 m1A dot blot assays

RNA was extracted from Huh-7 cells via the Trizol method. 10µL of RNA was denatured at 95°C for 3 min, immediately on ice for 2 min, and dropped onto the nitrocellulose membrane. Then, after air-drying, the specimens were dried at 120°C for 15 min. RNA not bound to the membrane was washed off with Wash Buffer (0.1% Tween-20 in 1×phosphate buffer saline (PBS)) for 3 min. At room temperature, the proteins were blocked with Blocking Buffer (5% Non-fat milk in Wash Buffer) for 1 h. The membranes were transferred to M1A antibody (Blocking Buffer1:2000 dilution) and cultured for 2 h at room temperature (or 4°C with shaking overnight). When the incubation was finished, at room temperature, the cells were washed four times with Wash Buffer for 5 min each. Subsequently, the washed nylon membrane was immersed in the secondary antibody solution (1:2000 dilution) and cultured for 1 h at room temperature. Then we washed the plates four times for 5 min each at room temperature with Wash Buffer. Finally, chemiluminescence was performed with an ECL chemiluminescence kit, and a chemiluminescence imaging system was used to record the chemiluminescence results.

### 2.13 Cell culture and transfection

The Huh-7 cell line (CL-0120, Procell) was cultured in DMEM supplemented with 10% fetal bovine serum (FBS) and 1% penicillin/streptomycin (PS) at 37°C with 5% CO_2_. The human monocytic leukemia cell line THP-1 (iCell-h188, iCell Bioscience) was procured from iCell Bioscience and cultured in Roswell Park Memorial Institute (RPMI) 1640 medium supplemented with 10% fetal bovine serum and antibiotics (penicillin/streptomycin). Cells were maintained in a humidified incubator with an atmosphere of 5% CO2 at a temperature of 37°C. THP-1 cells were treated with 100 nmol/L phorbol-12-myristate-13-acetate (P8139, Sigma) to induce macrophage differentiation for 48 h. The miR-4739 mimics (5’-AAGGGAGGAGGAGCGGAGGGGCCCU-3’ and 5’-UUCCCTCCUCCUCGCCUCCCCGGGA-3’) were transfected into Huh-7 cells using Lipofectamine™ 2000 (Invitrogen) transfection reagent. The upper chamber of the Transwell insert was used to culture Huh-7 cells overexpressing miR-4739, while the lower chamber was used to culture macrophages.

MiRNA inhibitors are chemically modified double-stranded oligonucleotides that can specifically bind to and inhibit endogenous miRNAs. MiR-4739 inhibitors are small molecule inhibitors targeting MiR-4739. In the presence of Lipofectamine™ 2000 (Invitrogen) transfection reagent, Huh-7 cells were transfected with miR-4739 inhibitor (5’-AGGGCCCCUCCGCUCCUCCUCCCUU-3’ and 5’- UCCCGGGGAGGCGAGGAGGAGGGAA-3’) and inhibitor NC (5’-UUCUCCGAACGUGUCACGUTT-3’ and 5’-ACGUGACACGUUCGGAGAATT-3’). On the day before transfection, we inoculated Huh-7 cells into plates of 96-well at 30×10^4^ cells/well. After 24 h, the fusion rate of cells reached 90%, and the cells were transfected with miR-4739 inhibitor and inhibitor NC. The transfection reagent and RNA were diluted in OPTI-MEM (31985062, Thermo) and incubated for 5 min at room temperature. Then, at room temperature, 50 μL of the diluted inhibitors and 50 μL of the diluted transfection reagent were mixed and incubated for 20 min. 100 μL of the incubated mixture was appended to the cell sample for continued incubation for 24 h.

### 2.14 Immunofluorescence

Immunofluorescence was used to detect the expression of secreted phosphoprotein 1 (SPP1) in macrophages co-cultured with Huh-7 cells. Briefly, macrophages were washed 3 times with PBS before fixation with 4% paraformaldehyde. Subsequently, macrophages were blocked with immunofluorescence sealing fluid (Beyotime) and incubated with anti-SPP1 (1:1000, sc-73631, Santa Cruz Biotechnology) at 4°C overnight. After washing with PBS, macrophages were incubated with a secondary Cy3-labelled goat anti-mouse IgG antibody (1:1000, A0521, Beyotime) for 1 h at room temperature. Finally, nuclei were stained with DAPI (Beyotime) for 10 min, and immunofluorescence was visualized under an immunofluorescence microscope.

### 2.15 Western blot

Following the manufacturer’s instructions, we extracted the total protein of Huh-7 cells, and the reagents were RIPA lysate buffer (Beyotime Biotechnology). After we measured the protein concentration via BCA protein assay, we used 10% SDS-PAGE gel to load and separate 40 μg of total protein and transferred it to polyvinylidene fluoride membranes. With 5% bovine serum albumin, the protein was further blocked, diluted in 0.05% Tris-buffered saline/Tween (TBST) and incubated with the primary antibody TRMT61A (1:1000, PA5-88013, Invitrogen), SPP1 (1:1000, PA5-34579, Invitrogen) or TRMT1 (1:1000, PA5-40929, Invitrogen) overnight at 4°C. The protein was cultured at room temperature with HRP-conjugated Goat Anti-Rabbit secondary antibody (1:5000, SA00001-2, Proteintech) for 2 h. After intensity analysis utilizing a Bio-Rad ChemiDoc XRS system (Bio-Rad, Hercules, CA, USA), we exposed the protein bands via an ECL kit (Millipore, St. Louis, MO, USA).

### 2.16 CCK-8 assay

In the presence of lipo2000 transfection regent, Huh-7 cells were transfected with miR-4739 inhibitor/NC. The cells were digested to make a single-cell suspension. Then, the cells were counted, and each group was diluted to 1×10^4^ cells/ml. Cells were inoculated into 96-well microplates (3599, Corning) at 1000 cells/well. We added 10 μL CCK-8 reagent to each well after the cells were fostered for 0 h, 24 h, 48 h, and 72 h, then cultured for 2 h. We used a microplate reader (Thermo, MA, USA) to analyze the absorbance at 450 nm. Absorbance can be used to express cell proliferation.

### 2.17 EdU assay

Cells were first infected with miR-4739 inhibitor and NC for 24 h. Then, before EdU (E607204, China) was appended, we inoculated the infected cells into the plates of 96-well (1 × 10^4^ cells/well) and cultured for 24 h. Based on the protocol, we incubated the cells for 2 h at 37°C and fixed them in 4% formaldehyde solution for 30 min, and then we added 150 μL of 2 mg/mL glycine for 5 min. Next, with 0.5% Triton X- 100 at room temperature, these cells were treated for 10 min. After, we utilized PBS to wash the cells and added 1× ApolloR reaction mix (100 μL/well), then treated these cells with EdU, and then at room temperature, we made them react for 30 min in the dark. After that, we added Hoechst 33342 (100 μL/well) for 30 min to visualize nuclei. Subsequently, we washed the plates with PBS, and then fluorescence microscopy (DM IL LED, Leica, Wetzlar, Germany) was utilized to observe the positive cells.

### 2.18 Transwell assay

We used 24-well plates (354480, BioCoat) to perform the transwell assay. The cells were first starved with 2% FBS-DMEM medium for 12 h and then suspended in DMEM medium without FBS before being appended to the upper chamber (1 × 10^5^ cells/well). At the same time, we added a DEME medium containing 10% FBS to the lower chamber. We used an incubator to incubate the plates for 72 h. Then, we incubated the cells, which migrated to the lower surface of the filter membrane. We fixed and stained these cells with 4% paraformaldehyde and 0.5% crystal violet. A cotton swab was gently used to scrape the cells that remained on the upper surface of the filter membrane. The inverted microscope captured the lower surfaces, and the counts were repeated three times.

### 2.19 Wound-healing assay

Without changing the medium, the monolayer was scratched, and the tip of a new 200-microliter gun tip was threaded through the center of the hole. After scraping, to remove the isolated cells, we gently washed them twice with PBS and then supplemented them with an excellent serum-free medium. Monolayer cells were photographed on a microscope at 0 h and 24 h, and wound spacing was measured.

### 2.20 Luciferase reporter assay

The RNAhybrid website analyzed the possible binding sites of miR-4739 in TRMT61A. Then, the recombinant luciferase reporter vector with mutation binding site was co-transfected into HEK293T cells with miR-4739 expression mimics using Lipofectamine 2000 (Invitrogen). Renilla luciferase expression vector psiCHECK-2 (Promega) was used as an internal reference. After 48 h of transfection, cells were harvested and lysed. Luciferase reporter assays were performed using the dual luciferase reporter assay system (Promega).

### 2.21 Statistical analysis

A t-test was used to compare the two groups, and *P* < 0.05 was considered statistically significant. All assays were repeated three times independently.

## 3 Results

### 3.1 The risk model identifies four RMRMs associated with the prognosis of patients with HCC

**Figure 1:**
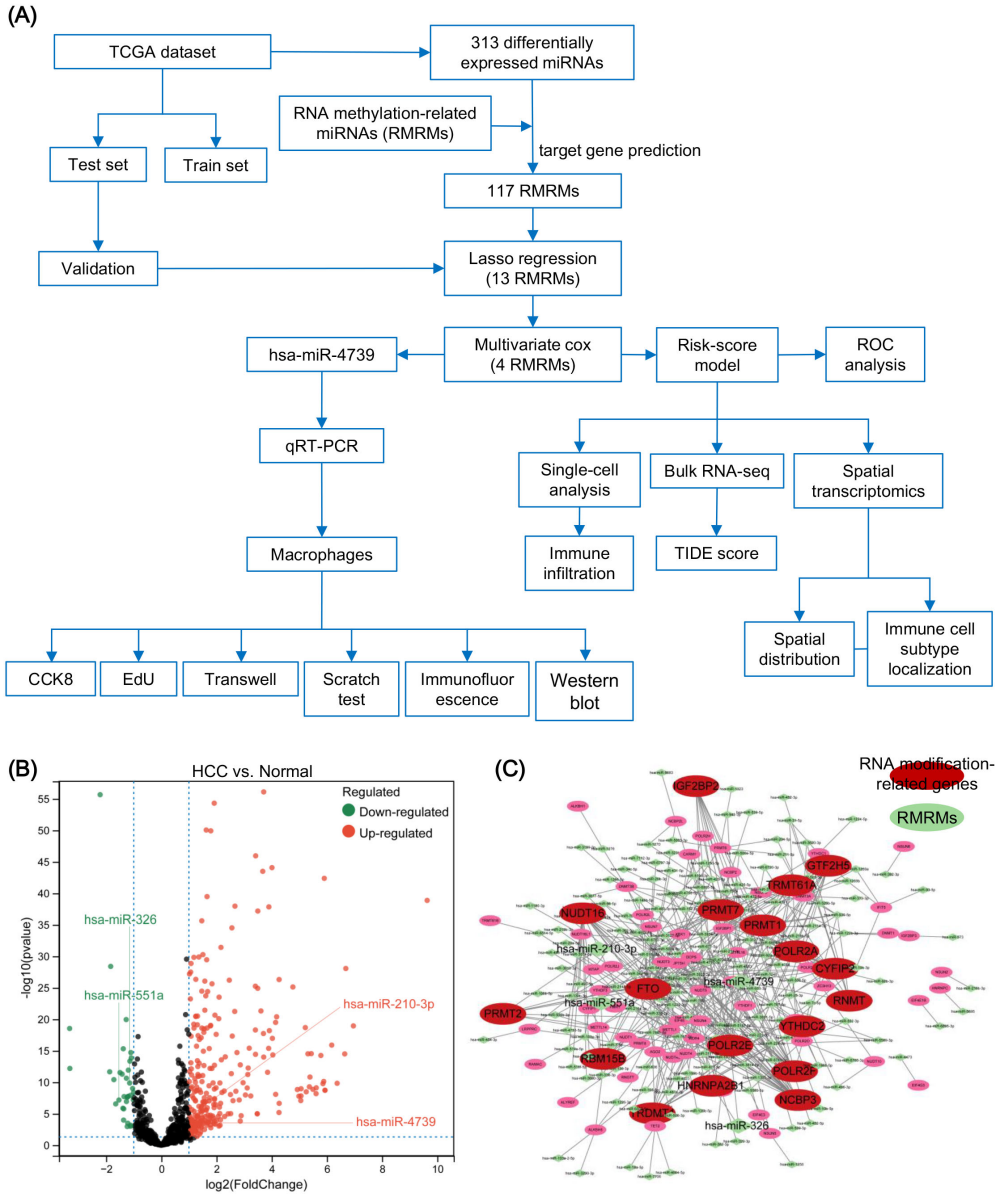
Identification of RNA methylation-related miRNAs (RMRMs) in hepatocellular carcinoma (HCC). **(A)** Flow diagram of the design of this study. **(B)** Differentially expressed miRNAs between samples derived from tumor and normal tissues through volcano map. **(C)** Regulatory network of miRNAs and m6A/m5C/m1A/m7G-associated genes. miRNAs are indicated in green; The m6A/m5C/m1A/m7G- associated genes are indicated in red.

**Figure 1A** illustrates the schematic construction and subsequent analysis of the RMRM risk model in HCC. We conducted a differential analysis for miRNAs between HCC and normal tissues. We found 313 differentially expressed miRNAs between HCC and normal tissues, including upregulated 279 and 34 downregulated differentially expressed miRNAs in HCC compared to normal tissues (**Figure 1B**). The target genes of differentially expressed miRNAs were predicted, and 117 miRNAs were found to be linked with RNA methylation-related genes (**Figure 1C**).

**Figure 2:**
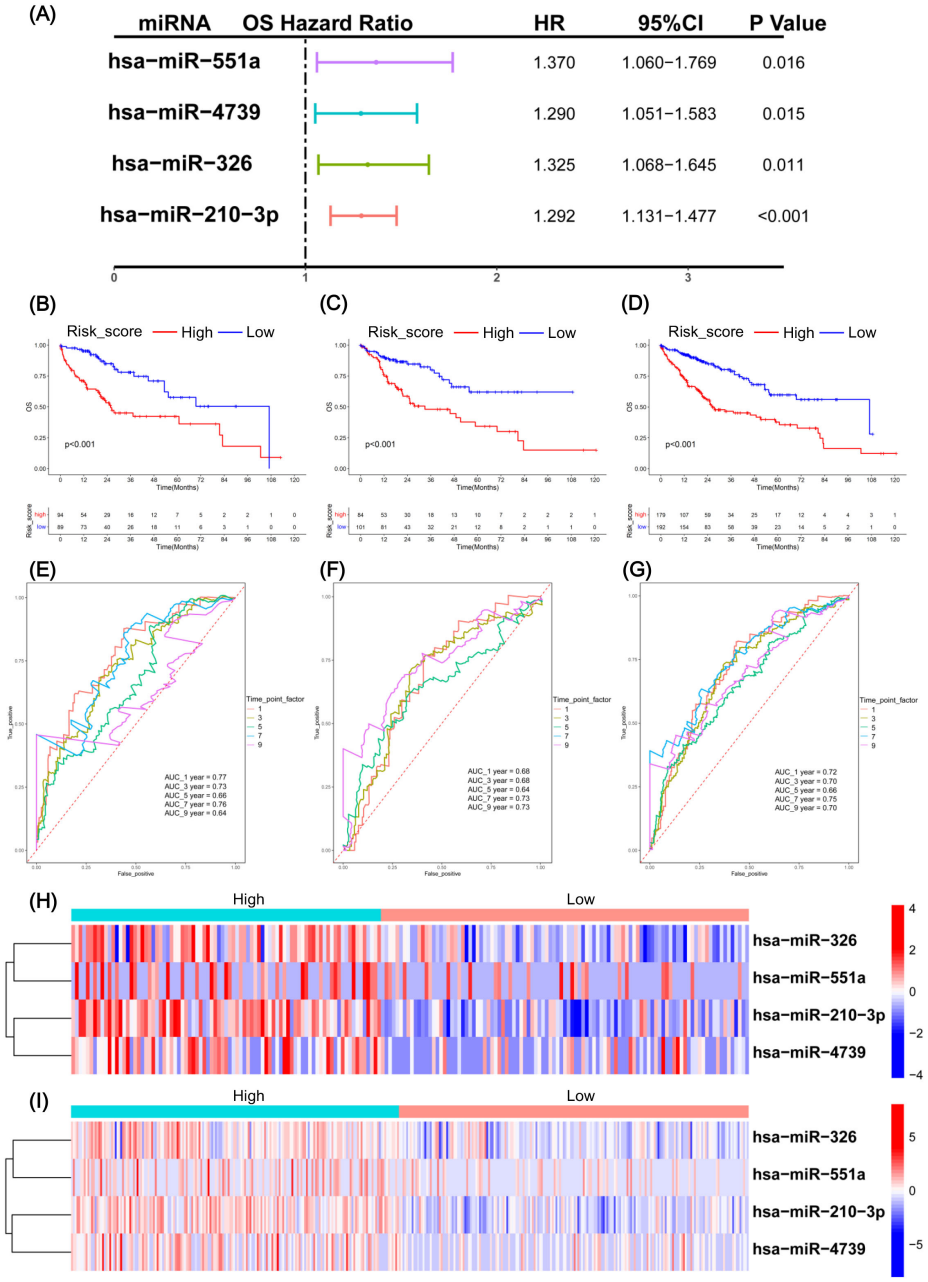
Construction and verification of RNA methylation-related miRNAs (RMRM) risk model in hepatocellular carcinoma (HCC). **(A)** Multivariate Cox regression analysis identified four RMRMs for risk model construction. **(B–D)** In the training set **(B)**, testing set **(C)**, and full data set **(D)**, the Kaplan-Meier (KM) survival curves were plotted between the groups with high and low risk. **(E–G)** ROC curves were plotted for the training set **(E)**, testing set **(F)**, and full data set **(G)** at 1, 3, 5, 7, and 9 years after diagnosis. **(H, I)** Heatmaps of selected four miRNAs in the testing set **(H)** and full data set **(I)**.

The miRNA data in TCGA-LIHC was divided into training and testing sets. The training set was used to construct the risk model. First, the univariate Cox analysis identified 28 RMRMs which were significantly correlated with HCC prognosis (*P* < 0.05) (**Supplementary Figure S1A**). Subsequent Lasso regression and multivariate Cox analyses further selected four RMRMs as prognostic markers, including miR-551a, miR-4739, miR-326, and miR-210-3p (**Figure 2A**, **Supplementary Figures S1B, C**). Next, we calculated the risk scores of 4 RMRMs in the testing set and plotted the KM and ROC curves. The risk score was as follows: Risk score = exp (0.2565 × miR-210-3p + 0.2818 × miR-326 + 0.2546 × miR-4739 + 0.3145 × miR-551a). The HCC samples in TCGA-LIHC were categorized into high- and low-risk groups. Compared to the overall survival (OS) of patients in the low-risk group, the patients in the high-risk group exhibited reduced OS, as validated in the training (*P* < 0.001) (**Figure 2B**), testing (*P* < 0.001) (**Figure 2C**), and total sets (*P* < 0.001) (**Figure 2D**). Subsequently, we focus the prognostic prediction of the RMRM model on the accuracy of predicting patient survival at 1, 3, 5, 7, and 9 years. The AUCs of the ROC curves in the training sets (**Figure 2E**), testing sets (**Figure 2F**), and total sets (**Figure 2G**) were indicated at 1, 3, 5, 7, and 9 years. The heatmap showed the expressions of miR-551a, miR-4739, miR-326, and miR-210-3p in the high- and low-risk groups in the testing and full data sets (**Figures 2H, I**, **Supplementary Figure S1D**). Furthermore, we also developed an integrated nomogram based on independent prognostic factors to calculate the individual OS for patients with HCC (**Supplementary Figure S1E**). This nomogram model exhibited superior predictive accuracy. The calibration plots confirmed their reliability in forecasting 3- and 5-year OS rates (**Supplementary Figure S1F**). Additionally, decision curve analysis (DCA) indicated that our integrated nomogram offered a significant net benefit over the risk score model and age alone (**Supplementary Figure S1G**). Collectively, these findings suggest that the nomogram provides a prognosis for patients with HCC.

### 3.2 ScRNA-seq profiling reveals risk score-associated immune landscape in HCC

**Figure 3:**
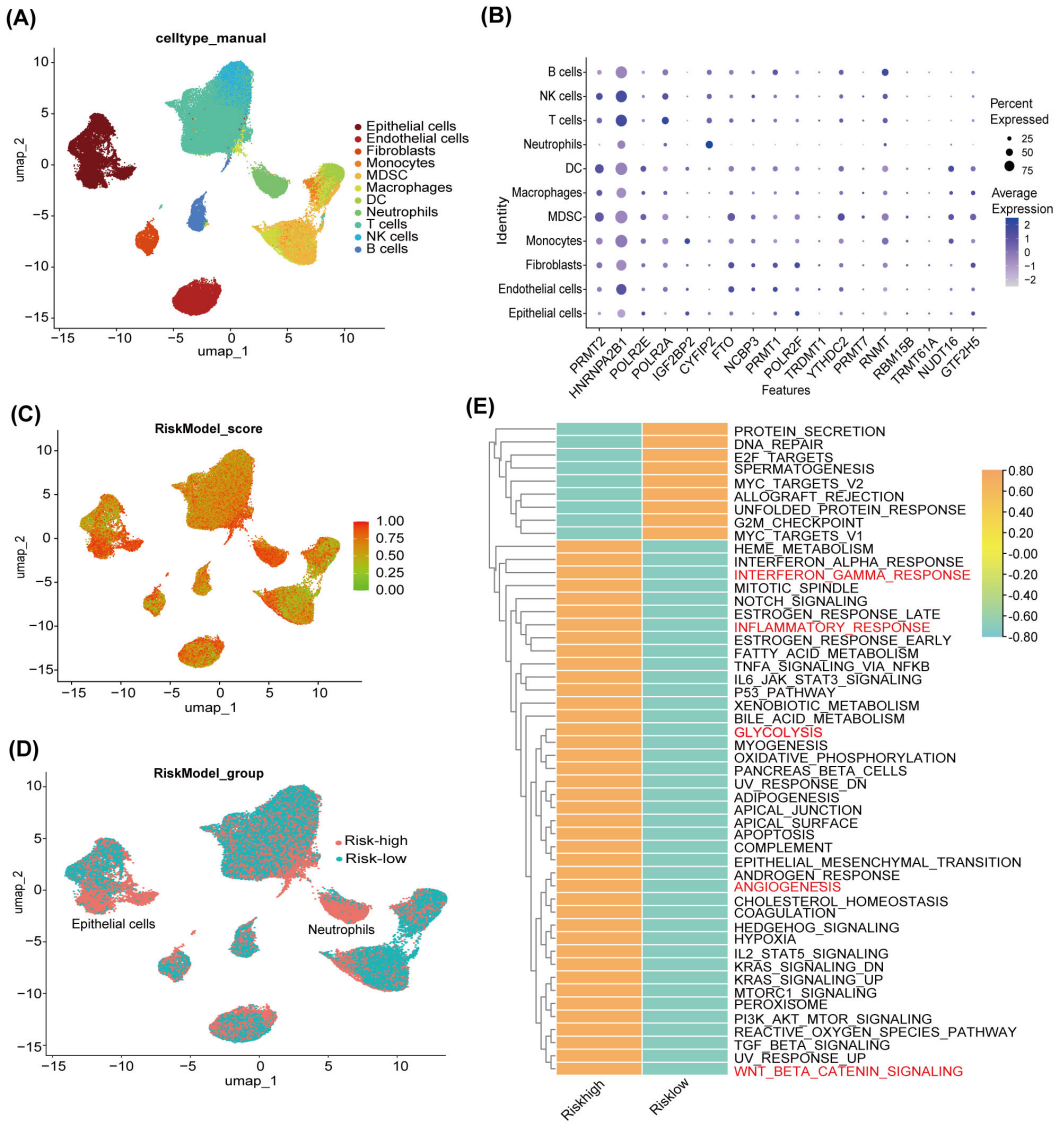
Assessment of RNA methylation-related miRNA (RMRM) risk score and its associated immune pathways at single-cell level. **(A)** The UMAP plots annotated cell types. **(B)** The expression profiles of 18 RNA modification-associated genes targeted by 4 RMRMs in various cell types. **(C)** The distribution of risk model scores across various cell types. **(D)** All cells were clustered into high-risk and low-risk groups. **(E)** GSVA of hallmark gene sets showed the pathways activated in high-risk and low-risk groups.

To further explore the heterogeneity of immune cells under the RMRMs risk model, we performed scRNA-seq analysis on 6 HBV-infected HCC (HBV-HCC) and 4 HBV tissues obtained from the GSE202642 dataset. After quality control, 98,904 cells from 10 patients were used for further analysis. The UMAP dimensionality reduction algorithm identified 11 distinct cell types: B cells, NK cells, T cells, neutrophils, dendritic cells (DCs), macrophages, myeloid-derived suppressor cells (MDSCs), monocytes, fibroblasts, endothelial cells, and epithelial cells (**Figure 3A**). Next, the expression profiles of 18 RNA modification-associated genes, which are the potential targets of miR-551a, miR-4739, miR-326, and miR-210-3p, constituting the risk model, were assessed in various cell types. The results revealed a differential expression pattern of these genes in different cells (**Figure 3B**). We developed a new risk-scoring algorithm to elucidate the relationship between the TCGA-derived risk model and the landscape of immune cells at the single-cell level. This approach ingeniously overcame the absence of miRNA expression data by employing GSVA on the expression of 18 RNA-modified genes targeted by the 4 miRNAs. Furthermore, we transformed the expression data of these target genes into a miRNA score according to the following formula: risk score = 1 - (x - min)/(max - min). The UMAP plot revealed the distribution of risk scores (**Figure 3C**), and cells were categorized into high-risk (pink) and low-risk (blue) groups (**Figure 3D**). GSVA showed that risk-high group enriched tumorigenesis and immune regulation pathways, including Interferon gamma response, Inflammatory response, Glycolysis, Angiogenesis, and Wnt beta catenin signaling (**Figure 3E**).

**Figure 4:**
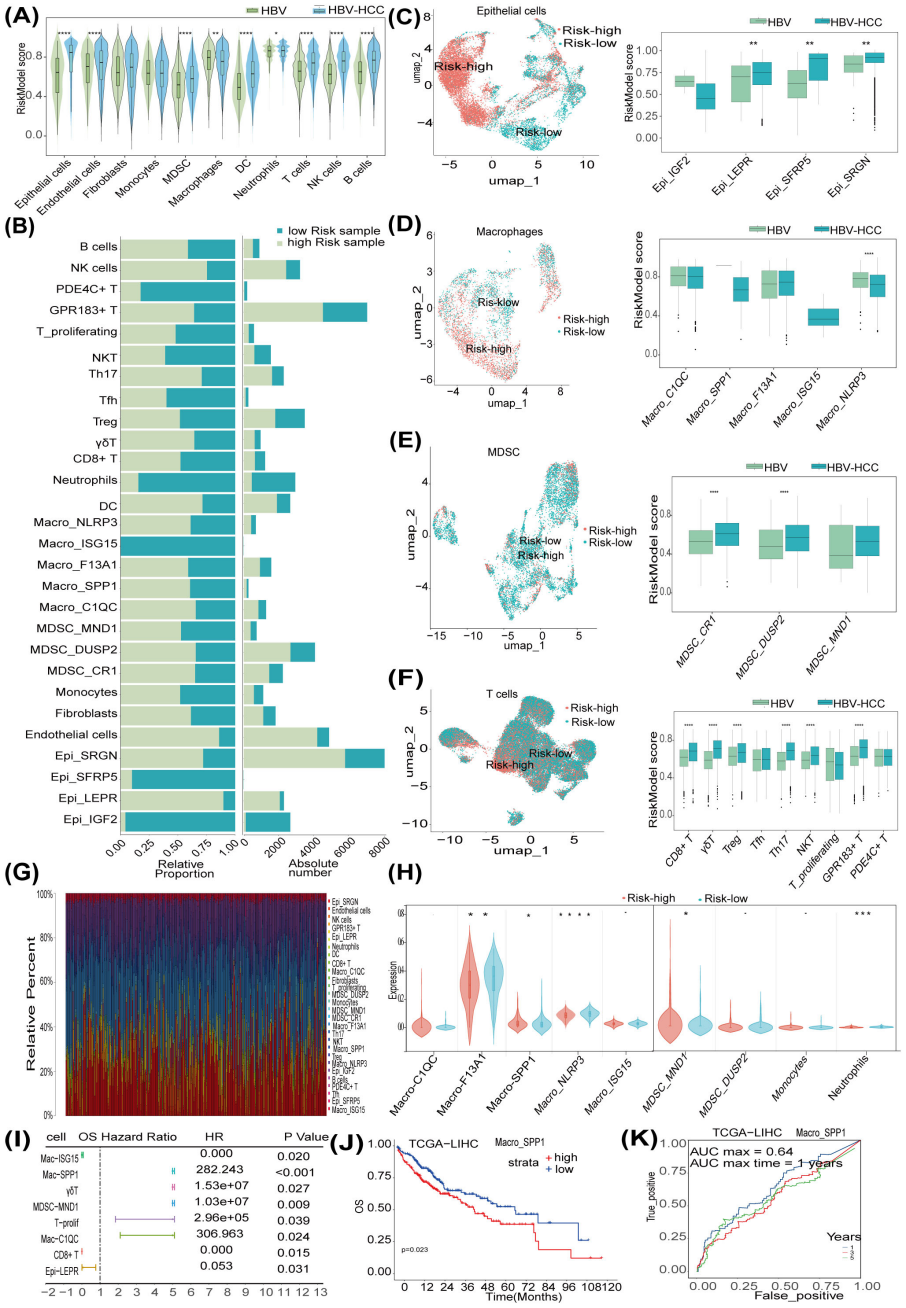
The immune regulatory landscape of RNA methylation-related miRNA (RMRM) risk score revealed by single-cell RNA sequencing (scRNA-seq) and bulk-seq analysis. **(A)** The violin plot showed the risk model score of different cell types between control and hepatocellular carcinoma (HCC) samples at the single-cell level. **(B)** The proportion of different cell subtypes in the high-risk and low-risk score groups. **(C-F)** Risk model score of cell subpopulation of epithelial cells, macrophages, T cells, and myeloid-derived suppressor cell (MDSC) cells using scRNA-seq data. **(G)** Infiltration analysis of tumor samples from TCGA-HCC bulk-seq, using the biomarker obtained from the scRNA-seq analysis. **(H)** The violin plot shows the infiltration of subtypes in risk-high and risk-low tumor samples. **(I)** Univariate Cox regression of prognosis-related immune cell subpopulation associated with RMRM risk score. **(J)** Kaplan-Meier (KM) survival curves show the association of secreted phosphoprotein 1 (SPP1)^+^ macrophage level with the OS survival of liver hepatocellular carcinoma (LICH) patients. **(K)** ROC curves were plotted for the diagnosis value of SPP1^+^ macrophage. *P < 0.05, **P < 0.01, ***P < 0.001, ****P < 0.0001.

Next, we performed a comparative analysis between HBV-HCC and HBV samples to evaluate the risk scores across various immune cell subtypes. As shown in **Figure 4A**, the risk score of epithelial cells, endothelial cells, MDSCs, DCs, neutrophils, B cells, NK cells, and T cells significantly increased in the HCC sample compared to the control. Subsequently, HCC samples were grouped based on the risk score of epithelial cells, with GSM6127499, GSM6127502, and GSM6127505 being the high-risk group and GSM6127500, GSM6127501, and GSM6127504 being the low-risk group (**Supplementary Figure S2A**). The high-risk group exhibited higher infiltration levels of immune cells than in the low-risk group (**Figure 4B**). We also noticed that the risk scores of endothelial cell subtypes, including Epi_LEPR, Epi_SERP5, and Epi_SRGN, in the HBV-HCC group were significantly higher than those in the HBV group (**Figure 4C**). The macrophage subtypes Macro_SPP1, Macro_F13A1, and Macro_C1QC increased in the high-risk group compared to the low-risk group, but their risk scores exhibited no statistical differences between the HBV-HCC and HBV groups (**Figures 4B, D**). MDSC subtypes, MDSC_CR1 and MDSC_DUSP2, showed higher risk scores in the HBV-HCC group compared to the HBV group (**Figure 4E**). Furthermore, compared to the low-risk and HBV groups, the high-risk and HBV-HCC groups demonstrated a higher percentage of T cell subtypes, such as regulatory T cells (Treg) and γδT cells (**Figures 4B, F**). Collectively, the above results illustrate the characteristics of immune cell subsets associated with risk scores in HCC.

Subsequently, we used samples from the TCGA-LIHC cohort to explore the correlation between the risk scores and immune cell subsets identified by scRNA-seq analysis. **Figure 4G** depicts the distribution of infiltration levels across various immune cell subtypes within HCC samples. The microsatellite instability (MSI) score was increased, while the TIDE score was decreased in the risk-high compared to the risk-low group (**Supplementary Figure S2B**). The risk-high group showed a higher level of Macro_F13A1 than the risk-low group (**Figure 4H**), which was consistent with the results in the scRNA-seq data (**Supplementary Figure S2C**). Univariate Cox regression analysis demonstrated that elevated infiltration levels of Macro-SPP1, MDSC-MND1, γδ T cells, T-proliferating cells, and Macro-C1QC were significantly correlated with an adverse prognosis of HCC patients, with the correlation of macrophages being the most significant, while the presence of CD8^+^ T cells and Macro-ISG15 was identified as a favorable prognostic factor (**Figure 4I**). KM survival analysis indicated that HCC patients with high levels of Macro-SPP1 and MDSC-MND1 exhibited significantly shorter OS times than those with lower infiltration levels (**Figure 4J**, **Supplementary Figure S2D**). ROC analysis validated the predictive accuracy of Macro-SPP1 and MDSC-MND1 (**Figure 4K**, **Supplementary Figure S2E**). Conversely, HCC patients exhibiting high levels of CD8^+^ T cells and Macro-ISG15 demonstrated favorable prognoses (**Supplementary Figures S2F-I**). In summary, these findings suggest the relationship between the risk scores of various cell subsets and the prognosis of HCC patients.

### 3.3 Spatial transcriptome shows risk score-related immune microenvironment structure

**Figure 5:**
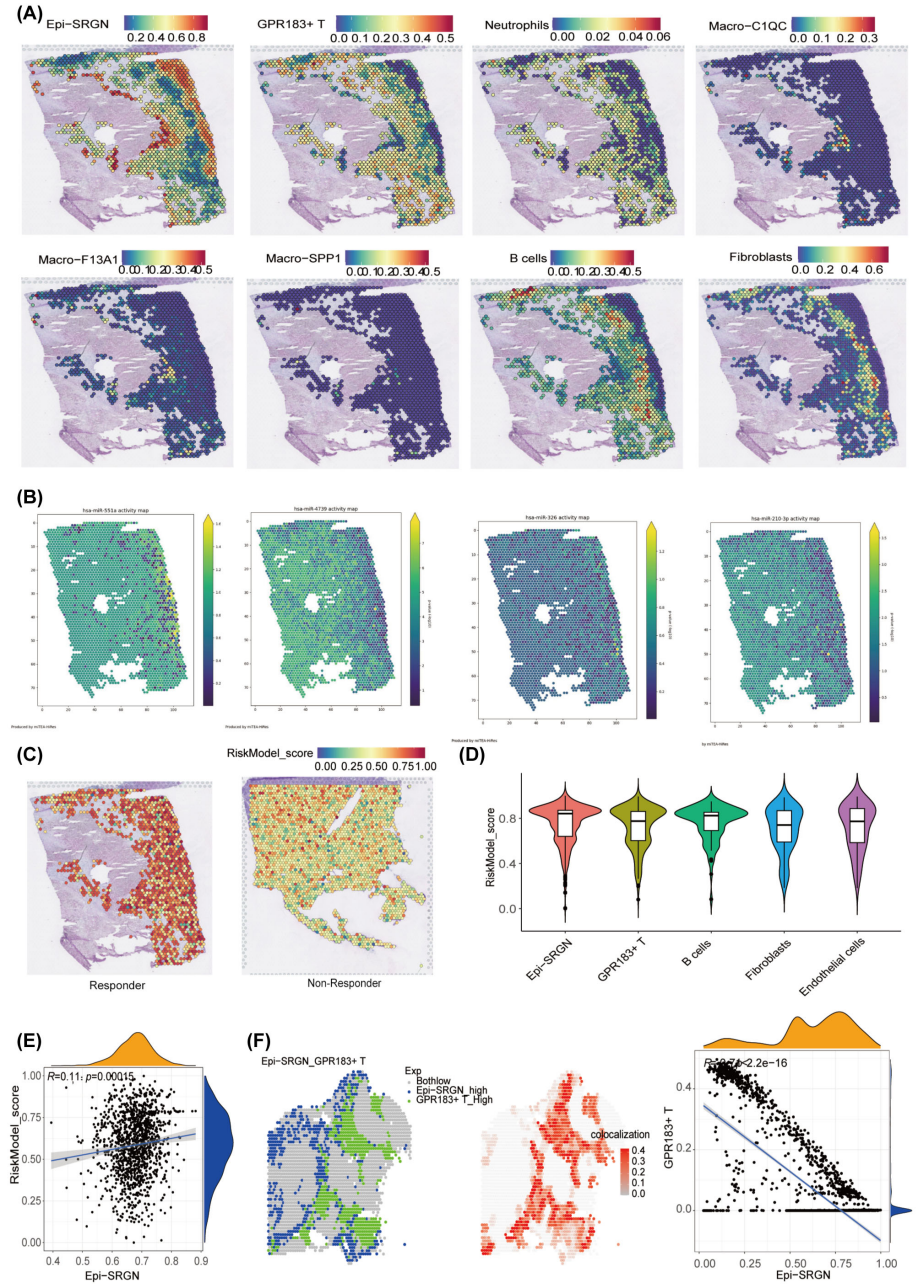
Spatial transcriptomics (ST) analysis showed the spatial structure of immune cells correlated with RNA methylation-related miRNA (RMRM) score. **(A)** Annotate the tissue sections by integrating single-cell RNA sequencing (scRNA-seq). Spatial mapping of cell subsets identified in the scRNA-seq data. **(B)** Spatial activity of four miRNAs. **(C)** Spatial mapping of the risk score in therapy responder and non-responder. Hepatocellular carcinoma (HCC) patients were treated with neoadjuvant cabozantinib, a multi-tyrosine kinase inhibitor, and nivolumab, a PD-1 inhibitor. **(D)** Differences in risk scores among cell subtypes identified by the spatial transcriptome. **(E)** The spatial relationship between the RiskModel and Epi−SRGN. **(F)** The spatial location correlation of Epi−SRGN and GPR183^+^ T cells.

To elucidate the spatial characteristics of risk score within the TME, we procured spatial transcriptome data of HCC (34) and integrated it with the scRNA-seq data. Leveraging the markers derived from scRNA-seq, we annotated the cells in spatial transcriptomic data into macrophage subtypes Macro-C1QC, Macro-F13A1, and Macro-SPP1, Neutrophils, GPR183^+^ T cells, epithelia subtypes Epi-SRGN, Epi-LEPR, Epi-IGF2, and Epi-SFRP5, Endothelial cells, Fibroblasts, and B cells (**Figure 5A**). We observed that Macro-C1QC and GPR183^+^ T infiltrated around Epi−SRGN and Epi−LEPR. Next, the spatial activities of 4 RMRMs (miR-551a, miR-326, miR-4739, and miR-210-3p) were evaluated using the miTEA method (**Figure 5B**). The results showed that Epi−SRGN regions exhibited spatial activities of 4 miRNAs. The spatial distribution of risk scores within the HCC microenvironment was also evaluated, and tumor regions exhibited notably high scores (**Figure 5C**). Patients who demonstrated a positive response to a multi-tyrosine kinase inhibitor, cabozantinib, and a PD-1 inhibitor, nivolumab, were presented with significantly higher scores than those who did not respond to the treatment (**Figure 5C**). Violin plots revealed that risk scores within the Epi−SRGN cell population were relatively elevated compared to other cell types (**Figure 5D**), leading us to select Epi-SRGN for in-depth analysis. The colocation analysis demonstrated a positive correlation between the position of risk scores and the location of Epi−SRGN and Epi−LEPR (**Figure 5E**, **Supplementary Figure S3A**) and a negative correlation between the location of Epi−SRGN and GPR183^+^ T cells (**Figure 5F**, **Supplementary Figure S3B**). In addition, a negatively correlated position relationship between Epi−SRGN cells and Macro−C1QC was found (**Supplementary Figure S3C**). Taken together, these results indicate the spatial relationship between risk scores and the location of immune cells.

### 3.4 Consensus clustering analysis based on the expression of 4 RMRMs

**Figure 6:**
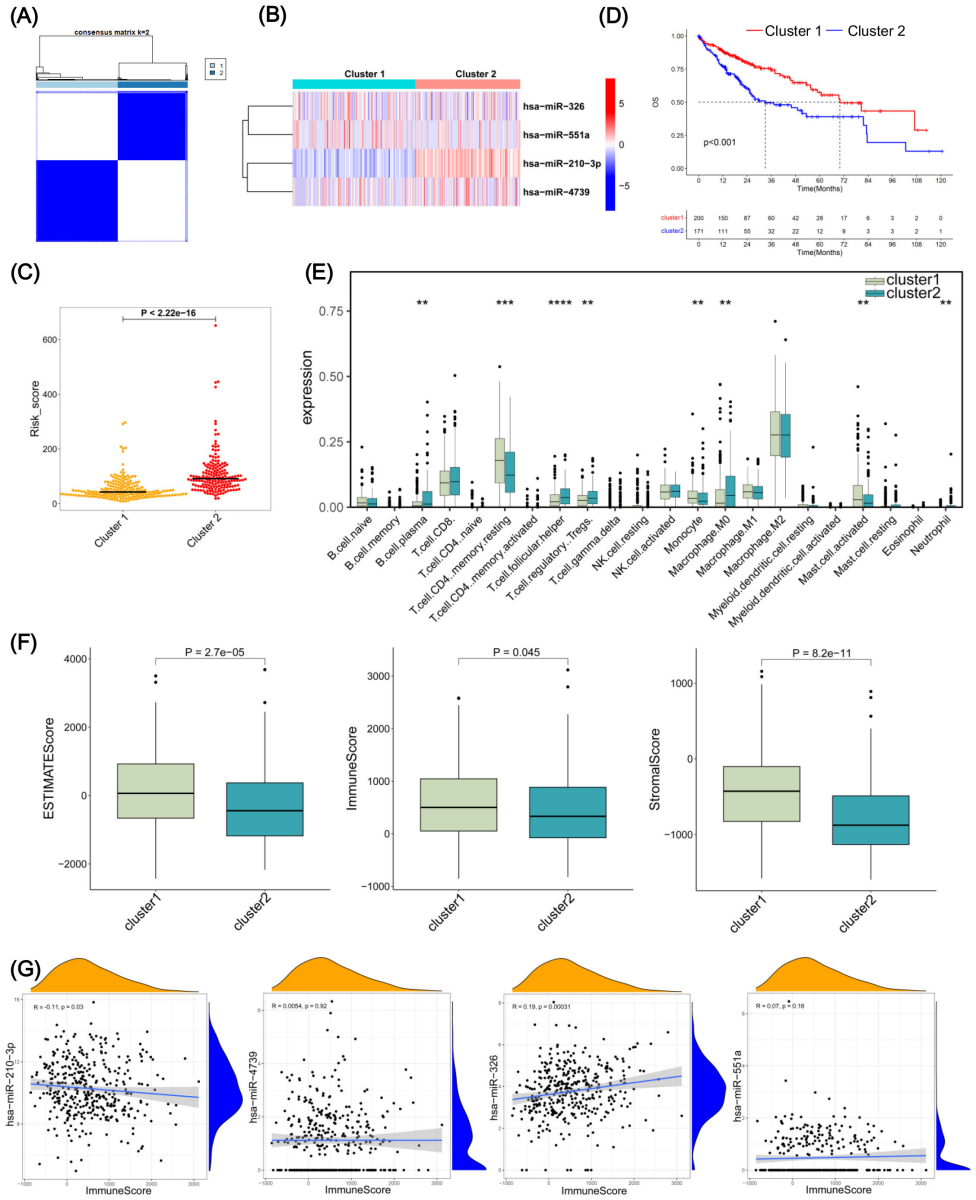
RNA methylation-related miRNA (RMRM) risk scores-related clustering in hepatocellular carcinoma (HCC) patients. **(A)** The consensus matrix. k=2. **(B)** Heatmaps of 4 RMRMs in the cluster 1 and cluster 2 groups. **(C)** Differential analysis of risk score between cluster 1 and cluster 2. **(D)** Kaplan-Meier (KM) survival analysis for cluster 1 and cluster 2. **(E)** Differential expressions of immune cell subpopulations in cluster 1 and cluster 2 are shown in the boxplot. **(F)** The correlations between RMRM risk score and immune infiltration. **(G)** The interrelation analysis between immune score and the expression of the four RMRMs. **P < 0.01, ***P < 0.001, ****P < 0.0001.

Consensus clustering was employed to classify HCC samples into distinct molecular subtypes based on 4 RMRMs expression patterns (**Figure 6A**). This unsupervised clustering method enhances the robustness of subtype identification by evaluating clustering stability across multiple iterations. Given the heterogeneity of HCC, identifying molecular subtypes helps to elucidate differences in tumor immune microenvironment characteristics, prognostic implications, and potential therapeutic responses. We found that the optimal clustering results were produced after choosing k=2 (**Figure 6A**). The heatmap showed the expression profiles of 4 RMRMs in cluster 1 and cluster 2 (**Figure 6B**). Cluster 2, exhibiting a higher risk score, showed a worse prognosis than cluster 1 (**Figures 6C, D**). Next, we further explored the immune infiltration characteristics of cluster 1 and cluster 2. The comparative analysis between 2 clusters highlighted a pronounced reduction in the infiltration of regulatory T cells (Tregs), plasma B cells, follicular helper T cells, and M0 macrophage in cluster 1 compared to cluster 2, while the infiltration of monocyte, activated mast cell, CD4 memory resting T cells were drastically increased (**Figure 6E**). The ESTIMATE, immune, and stromal scores in cluster 1 were higher than in cluster 2 (**Figure 6F**). We also observed that miR-210-3p was negatively correlated with the ESTIMATE, immune, and stromal scores, while miR-326 exhibited an opposite trend (**Figure 6G**, **Supplementary Figures S4A, B**). Subsequently, we performed a differential analysis between cluster 1 and cluster 2. We found 147 DEGs between cluster 1 and cluster 2, including 94 upregulated and 53 downregulated DEGs in cluster 1 compared to cluster 2 (**Supplementary Figure S4C**). GO enrichment analysis found that upregulated DEGs were enriched in mitosis-related pathways, such as mitotic nuclear division, mitotic cell cycle phase transition, regulation of mitotic metaphase/anaphase transition, and mitotic spindle assembly checkpoint signaling (**Supplementary Figure S4D**). KEGG enrichment analysis revealed that upregulated DEGs were associated with cellular senescence, p53 signaling pathway, HIF-1 signaling pathways, glycolysis/gluconeogenesis, viral carcinogenesis, and Foxo signaling pathway (**Supplementary Figure S4E**). Our results demonstrated that the identified subtypes exhibited significant differences in immune infiltration and survival outcomes, supporting the biological relevance of the classification.

### 3.5 TMB and drug sensitivity analyses of cluster 1 and cluster 2

**Figure 7:**
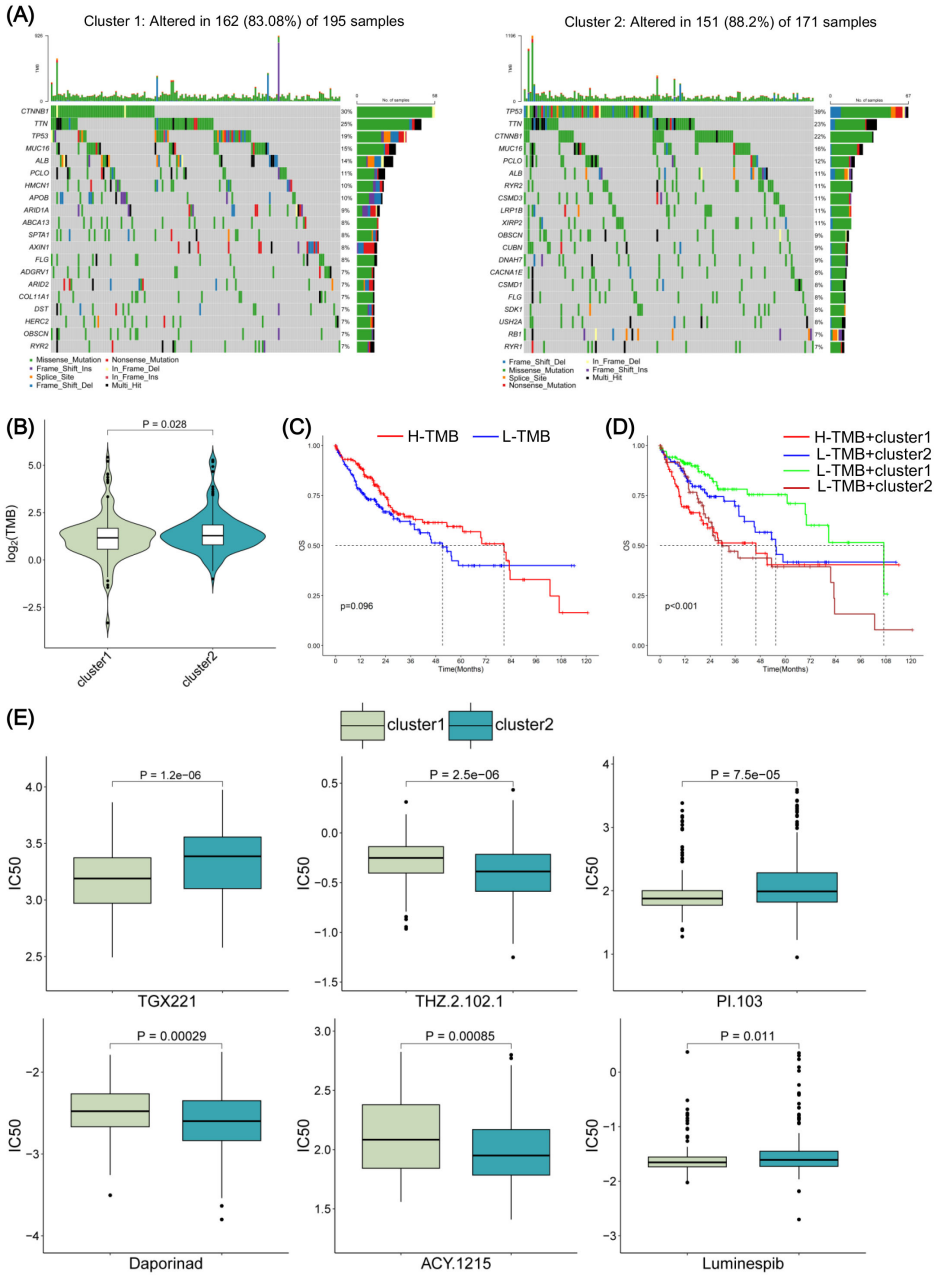
Tumor mutation burden (TMB) and drug sensitivity analyses of cluster 1 and cluster 2. **(A)** According to the mutation proportion, the waterfall chart was drawn to demonstrate the mutation type and rate of twenty genes in RNA methylation-related miRNA (RMRM) risk score-related clusters. **(B)** TMB differences analysis between cluster 1 and cluster 2. **(C)** Kaplan-Meier (KM) survival curves between the groups with high and low TMB. **(D)** KM survival curves between diverse levels of TMB combined with the cluster group. **(E)** Drug sensitivity analysis between risk score-related clusters.

We then obtained mutation data of TCGA-LIHC samples to analyze TMB in cluster 1 and cluster 2. The proportion of mutations in cluster 2 was higher than in cluster 1 (**Figure 7A**), and the mutation load index TMB index was overall higher in cluster 2 than in cluster 1 (**Figure 7B**). The OS rate of ten years between patients with high and low TMB showed no statistical significance (**Figure 7C**). However, the OS rate of cluster 1 and cluster 2 in patients with high and low TMB were significantly correlated with the prognosis (*P* < 0.001) (**Figure 7D**). To evaluate the relationship between drug sensitivity and consensus clusters, we compared IC50 values of different drugs between cluster 1 and cluster 2 (**Figure 7E**). Specifically, we obtained the IC50 values of samples from the TCGA-LIHC database. Based on unsupervised consensus clustering analysis, we compared the IC50 values between the two groups and found significant changes in the IC50 values of six drugs. The IC50 values of TGX221, PI.103, and Luminespib in cluster 1 were significantly lower than in cluster 2. Conversely, cluster 1 exhibited an increase in the IC50 values of THZ.2.102.1, Doparinad, ACY.1215 when compared to cluster 2. These findings indicate the differences in TMB and drug sensitivity between cluster 1 and cluster 2.

### 3.6 MiR-4739 is involved in the regulation of HCC by targeting TRMT61A

**Figure 8:**
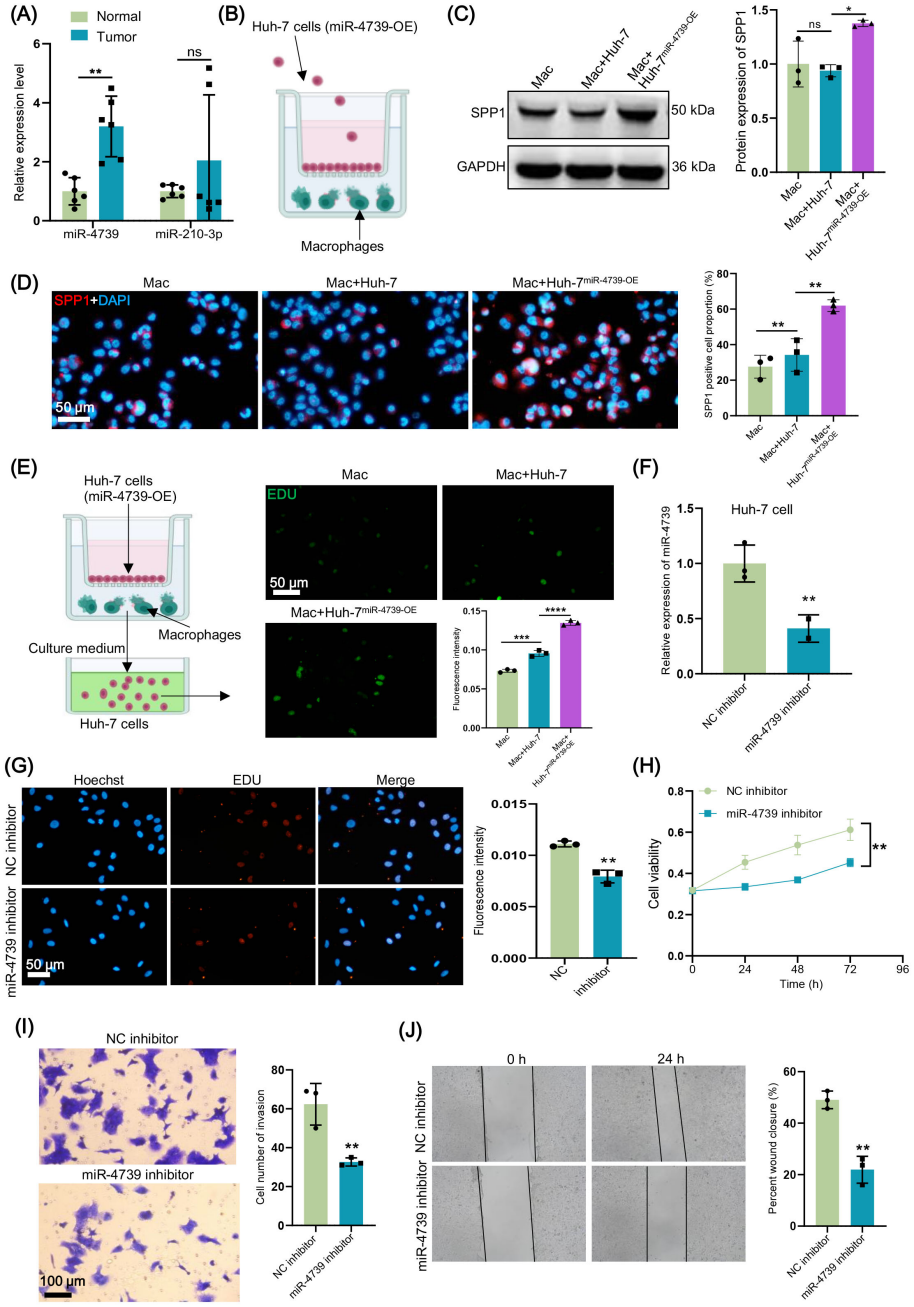
miR-4739 in hepatocellular carcinoma (HCC) cells induces secreted phosphoprotein 1 (SPP1)^+^ macrophages to drive HCC cell proliferation. **(A)** The relative expression level of miR-4739 and miR-210-3p in HCC tumor and normal paracancerous tissues was detected through qRT-PCR. **(B)** Graphical representation of the co-culture of HCC cell line Huh-7 cells and THP-1 induced macrophages. **(C)** The expression of SPP1 in macrophages co-cultured with miR-4739-overexpressed Huh-7 cells was detected by western blot. **(D)** Immunofluorescence showing the SPP1 positive macrophages after co-culture with Huh-7 cells with different treatments. **(E)** EdU detected cell proliferation of HCC cells after adding the culture medium of macrophages induced by Huh-7 cells with different treatments. **(F)** qRT-PCR was used to verify the knockdown effect of miR-4739 inhibitor in Huh-7 cells. **(G, H)** CCK8 **(G)** and EdU **(H)** detected cell proliferation of Huh-7 cells. **(I, J)** The invasion and migration of Huh-7 cells were detected by transwell **(I)** and scratch **(J)** experiments. ns = no significance, *P < 0.05, **P < 0.01, ***P < 0.001, ****P < 0.0001.

Considering the most significant correlation between Macro-SPP1 and the poor prognosis of HCC patients and the higher risk score exhibited by Macro-SPP1, we explored the impact of RMRMs in HCC cells on *SPP1* ^+^ macrophages. Compared to the normal paracancerous tissues, miR-4739 and miR-210-3p were significantly upregulated in HCC tumor tissues in the miRNA-seq data (**Table 2**), and qRT-PCR analysis further confirming the significant upregulation of miR-4739 (**Figure 8A**). Thus, we selected miR-4739 for subsequent analysis. Huh-7 cells were transfected with miR-4739 mimics and co-cultured with THP-1-induced macrophages for 24h (**Figure 8B**). As expected, overexpression of miR-4739 in Huh-7 cells significantly increased the expression of *SPP1* in macrophages and the proportion of *SPP1* ^+^ macrophages (**Figures 8C, D**). We noticed that the conditioned media derived from macrophages stimulated by Huh-7 cells with miR-4739 overexpression was found to enhance the proliferative capacity of Huh-7 cells (**Figure 8E**), suggesting an underlying interaction between *SPP1* ^+^ macrophages and Huh-7 cells. Additionally, we investigated the direct effects of miR-4739 on cancer cells. Considering the high expression of miR-4739 in cancer cells, we knocked down miR-4739 in Huh7 cells using specific miR-4739 inhibitors (**Figure 8F**) and observed reduced proliferation (**Figure 8G**), cell viability (**Figure 8H**), invasion (**Figure 8I**), and migration of Huh-7 cells (**Figure 8J**) compared to the NC inhibitor group. Together, these results demonstrate the critical role of miR-4739 in malignant features in HCC.

**Table 2:** RMRM expression levels in miRNA-seq data.

miRNA	log2Fold Change	Adjust *P* values	Regulation
miR-210-3p	1.03	2.47×10^-5^	Up
miR-326	-1.15	1.35×10^-10^	Down
miR-4739	1.42	2.92×10^-4^	Up
miR-551a	-1.54	6.80×10^-8^	Down

**Figure 9:**
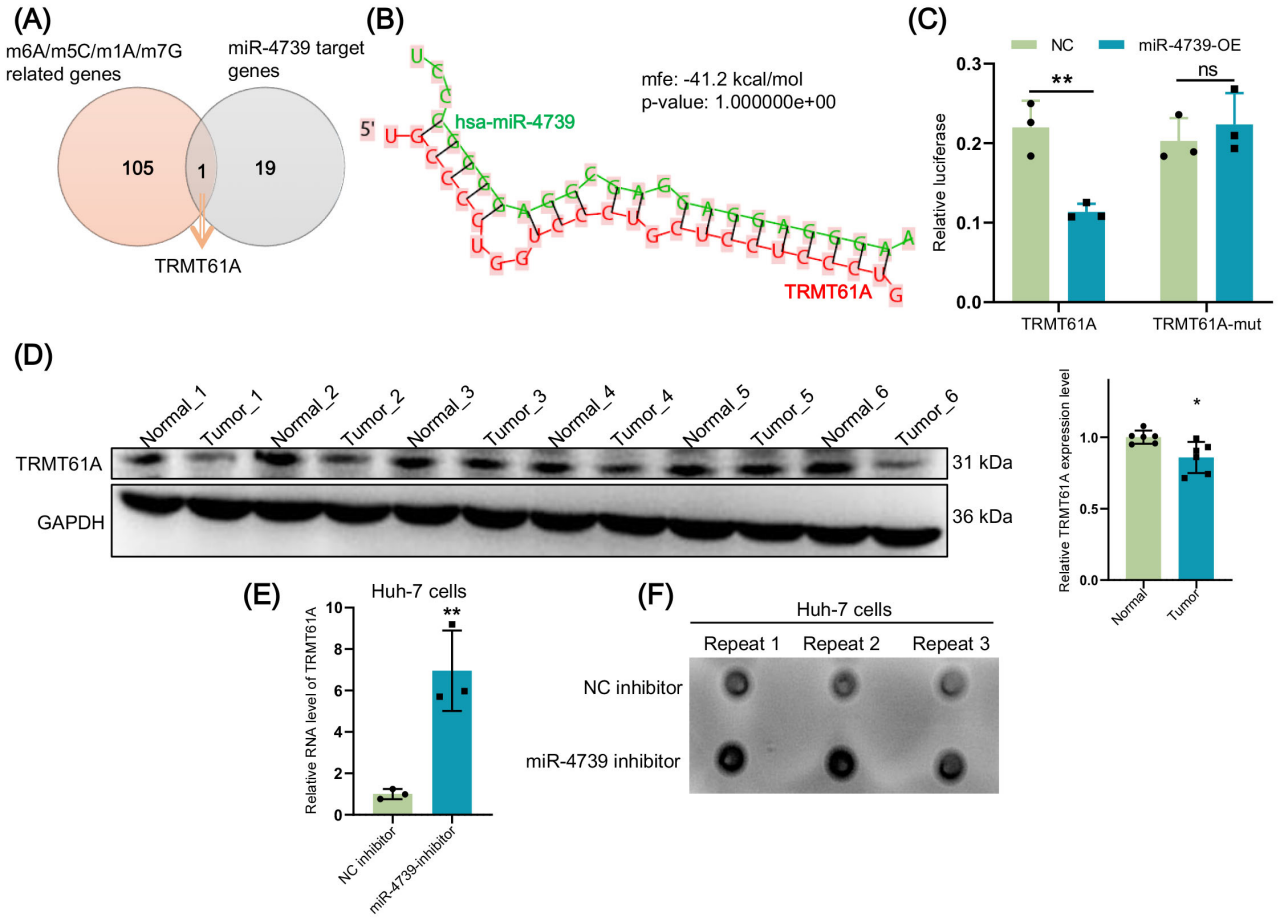
miR-4739 targets and inhibits TRNA methyltransferase 61A (TRMT61A) to suppress m1A methylation. **(A)** Venn diagram shows the number of overlapping gene *TRMT61A* in m6A/m5C/m1A/m7G-associated genes and miR-4739 target genes. **(B)** RNAhybrid predicted sequence complementarity maps of miR-4739 and 3’ UTR of TRMT61A. **(C)** Dual-luciferase reporter assay detected the targeting relationship of miR-4739 with TRMT61A 3’ UTR. **(D)** The expression of TRMT61A was detected through western blot in the hepatocellular carcinoma (HCC) tumor and normal paracancerous groups. **(E)** After inhibiting miR-4739, the expression of *TRMT61A* in Huh-7 cells was detected through qRT-PCR. **(F)** Dot blot assay measured the m1A modification after knocking down miR-4739 in Huh-7 cells. ns = no significance, *P < 0.05, **P < 0.01.

To clarify how miRNA regulates a specific RNA modification to mediate the biological processes of HCC cells, we overlapped m6A/m5C/m1A/m7G-related genes with miR-4739 target genes and identified *TRMT61A* (**Figure 9A**). In fact, there was a predictive binding site between miR-4739 and *TRMT61A* (**Figure 9B**), and this prediction was confirmed by the dual-luciferase reporter assay (**Figure 9C**). Western blot analysis showed that the expression of *TRMT61A* was significantly reduced in clinical HCC tumor samples compared to the normal paracancerous tissues (**Figure 9D**). However, miR-4739 inhibition led to a significant increase in the expression level of *TRMT61A* in Huh-7 cells (**Figure 9E**). We used the dot blot assay to examine the impact of miR-4739 on m1A modification, and the results demonstrated that miR-4739 inhibition promoted m1A modification in Huh-7 cells (**Figure 9F**). These findings suggest that miR-4739 may regulate the progression of HCC by inhibiting TRMT-mediated m1A modification.

## 4 Discussion

RNA methylation is pivotal in RNA transcription, splicing, and translation (35) and is closely associated with immune cell infiltration in cancer (36). This study established a novel prognostic model of RMRMs for HCC. ScRNA-seq data integrated spatial transcriptomes revealed various immune cell subtypes associated with risk models and delineated potential relationships between risk scores and the spatial location of these subtypes within tissues. Moreover, miR-4739 upregulation in HCC cells remarkably induced *SPP1* ^+^ macrophage differentiation. This phenomenon may be attributed to the fact that miRNA-4739 targets and suppresses *TRMT61A*, thereby promoting the malignant characteristics of HCC cells, which subsequently induces the differentiation of *SPP1* ^+^ macrophages.

This study established a risk model of RNA-methylation miRNAs for HCC. MiRNAs are diagnostic markers for HCC (37). A growing body of evidence has revealed significantly abnormal RNA methylation levels and the dysregulation of enzymes related to RNA methylation in HCC tissues and cell lines (38). However, a few studies have focused on using miRNAs associated with RNA methylation modifications to predict HCC. Previous studies have demonstrated a crosstalk between miRNA and RNA methylation in HCC (39). Our study also validated that RNA methylation-related genes were the target genes of differentially expressed miRNA between HCC and normal samples. Subsequently, these RMRMs were used to construct prognostic risk models. After pre-screening calculations, we identified 4 miRNAs associated with the prognosis of HCC, including miRNA-551a, miRNA-4739, miRNA-326, and miRNA210-3p. A previous study reported that increased expression of microRNA 551a blocks breast tumorigenesis (40). MiR-4739 serves as a biomarker of doxorubicin chemoresistance in breast cancer, with its overexpression facilitating the proliferation, progression, and survival of cancer cells (41). MiRNA-326 regulates the progression of various cancers (42–44) and has diagnostic and prognostic roles in HCC (45). These findings highlight the reliability of the four RMRMs in the prognostic prediction of HCC. In this study, HCC samples in TCGA-LIHC could be categorized into high- and low-risk groups based on the expressions of 4 RMRMs. ScRNA-seq and spatial transcriptomics data also validate the effectiveness of this prognostic risk model. Specifically, scRNA-seq analysis revealed differences in risk scores among various immune cell subtypes in HBV-HCC and HBV tissues, with the high-risk group significantly enriched in tumorigenesis and immune regulation pathways, indicating that the risk model effectively distinguishes risk characteristics of different immune cell subtypes. Spatial transcriptomics further validated the spatial distribution of risk scores within the tumor microenvironment, showing that high-risk score regions highly overlapped with tumor areas and were positively correlated with the spatial localization of specific cell types (such as Epi-SRGN), confirming the effectiveness of the risk model at the spatial level. RMRMs should be considered for better disease management.

Our results revealed the relationship of the prognostic risk model of RMRMs with the immune response. RNA methylation-miRNAs have been demonstrated to play a critical role in immune response (46). Our study showed differences in the risk scores of RMRMs of various immune cells between HCC and control samples. Furthermore, we also found the spatial correlation among the RMRMs risk score, tumor cells, and immune cell subtypes. In this study, we discovered that *SPP1* ^+^ macrophages were significantly increased in the high-risk group of the RMRMs and were positively correlated with poor prognosis. *SPP1* ^+^ macrophages represent an emerging subset of macrophages and are implicated in tumorigenesis. A study has indicated that macrophage polarity, defined by the expression of *CXCL9* and *SPP1* rather than traditional M1 and M2 markers, controls human cancers (47). *SPP1* ^+^ macrophages interacted with CAFs, forming a tumor immune barrier restricting immune cell infiltration into the tumor core, thus confining anti-PD-1 therapy in HCC mice (48). In our results, the overexpression of miR-210-3p in HCC cells induced *SPP1* ^+^ macrophages, promoting cancer cell proliferation. These data underscore the significance of *SPP1* ^+^ macrophages in the malignancy of HCC and their interaction with RNA epigenetic modification-related miRNAs in modulating the tumor microenvironment.

This study validated that miR-4739 promoted HCC progress and bond to *TRMT61A*. The noncatalytic subunit of tRNA methyltransferase 6 (*TRMT6*) forms a tRNA methyltransferase complex with TRMT61A to catalyze the methylation of m1A (49). A related study has shown that the m1A methyltransferase formed by *TRMT6* and *TRMT61A* was inversely correlated with HCC survival (23). Our findings indicated lower protein levels of *TRMT61A* in HCC tissues than in the normal samples. *TRMT61A* has been explored in cancers. For example, the expression of *TRMT61A* was significantly upregulated in bladder cancer cell lines compared to SV-HUC-1 cells (50). However, the depletion of *TRMT6/TRMT61A* markedly impaired the proliferative capacity of bladder cancer cell lines. In contrast, our *in vitro* experiments demonstrated that miRNA-4739 inhibit *TRMT61A* expression, and miRNA-4739 promoted the proliferation of Huh-7 cells and enhanced their invasive and migratory capabilities. These results suggested *TRMT61A* inhibition may promote HCC development.

## 5 Conclusion

In conclusion, our findings highlighted a novel RMRM risk model associated with the immune microenvironment and prognosis of HCC. ScRNA-seq data integrated spatial transcriptomics unveiled a diverse array of immune cell subtypes linked to the RMRMs risk model, shedding light on the spatial distribution of these subtypes within tissues and their correlation with risk scores. Furthermore, we observed that the upregulation of miR-4739 in HCC cells significantly induced *SPP1* ^+^ macrophages, thereby promoting cancer cell proliferation. We also found that miR-4739 mediated m1A methylation by inhibiting *TRMT61A.* These findings provide a new perspective on the spatial interplay between RNA epigenetic modifications and immune cell subsets within the tumor microenvironment, offering novel molecular targets for HCC immunotherapy.

## Data Availability

The datasets presented in this study can be found in online repositories. The names of the repository/repositories and accession number(s) can be found in the article/**Supplementary Material**.

## Supplementary material

Supplementary Figure 1 Establishment of a nomogram model integrating risk score and clinical information. **(A)** The forest plot showed the Univariate Cox regression of 28 prognosis-related RNA methylation-related miRNAs (RMRMs). **(B)** The analysis of Lasso regression screened thirteen key RMRMs. **(C)** Cross-validation in the study of Lasso regression for tuning parameter selection. **(D)** Heatmaps of selected four miRNAs in the training set. **(E)** The calibration plots confirmed the reliability of the nomogram. **(F)** A nomogram predicting the 3- and 5-year OS for patients with hepatocellular carcinoma (HCC). **(G)** Decision curve analysis (DCA) indicates the nomogram offered a significant net benefit over the risk score model and age.

Supplementary Figure 2 Immune characteristics of cell subsets associated with the risk model. **(A)** The risk model score in each sample in the single-cell RNA sequencing (scRNA-seq) dataset. **(B)** The correlation between immune infiltration and risk model score in TCGA- hepatocellular carcinoma (HCC). **(C)** Differential infiltration analysis of T cell subtypes between high- and low-risk model score groups. **(D)** Kaplan-Meier (KM) survival curves show the association of myeloid-derived suppressor cell (MDSC)- meiotic nuclear divisions 1 (MND1) level with the OS survival of liver hepatocellular carcinoma (LICH) patients. **(E)** ROC curves show the diagnosis value of MDSC-MND1. **(F)** KM survival curves show the association of the Mcro-ISG15 level with the OS survival of LICH patients. **(G)** ROC curves show the diagnosis value of Mcro-ISG15. **(H)** KM survival curves show the CD8^+^ T cell level association with the OS survival of LICH patients. **(I)** ROC curves showing the diagnosis value of CD8^+^ T cell.

Supplementary Figure 3 Spatial position relationship between risk score, immune cells, and epithelial cells. **(A)** Spatial correlation between risk score and Epi-LEPR cell. **(B)** Spatial correlation between Epi-SRGN cell and GPR183^+^ T cell. **(D)** Spatial correlation between Macro-C1QC cell and GPR183^+^ T cell.

Supplementary Figure 4 Differential analysis for RNA methylation-related miRNA (RMRM)-related clusters. **(A, B)** The interrelation analysis between the ESTIMATE score and the expression of the four RMRMs **(A)** and between the stromal score and the expression of the four RMRMs **(B)**. **(C)** The volcano plot shows the differentially expressed genes (DEGs) between cluster 1 and cluster 2. **(D)** GO enrichment analysis of upregulated DEGs between cluster 1 and cluster 2. **(E)** KEGG enrichment analysis of upregulated DEGs between cluster 1 and cluster 2.
