# Supplementary figures and images for: Single-cell and spatial transcriptomics reveal correlation between RNA methylation-related miRNA risk model and immune infiltration in hepatocellular carcinoma
Source: Front Oncol. 2025 May 9;15:1553239. doi: 10.3389/fonc.2025.1553239 (PMC12098086; doi:10.3389/fonc.2025.1553239)

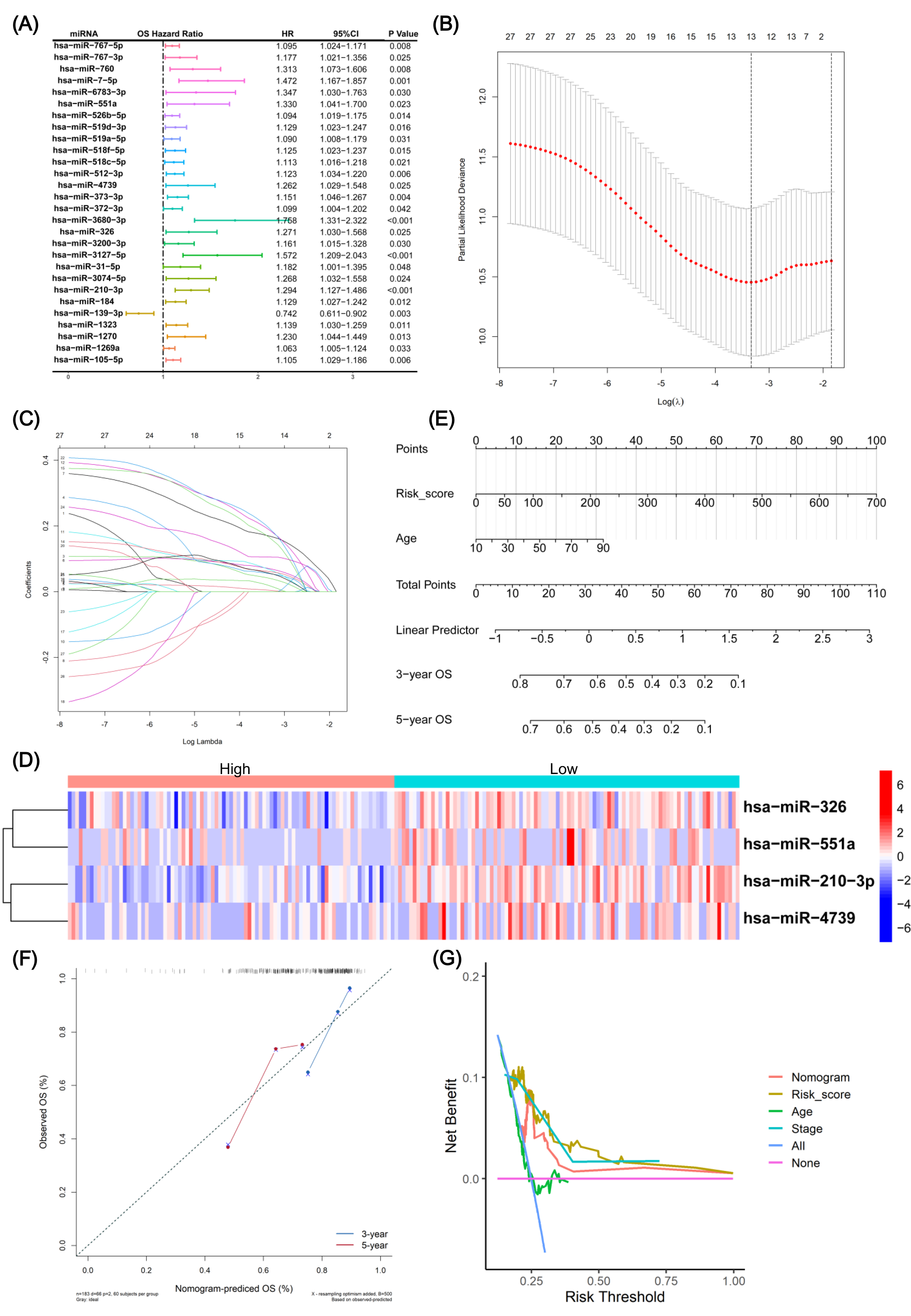

Supplement: Supplementary Figure 1 — Establishment of a nomogram model integrating risk score and clinical information. (A) The forest plot showed the Univariate Cox regression of 28 prognosis-related RNA methylation-related miRNAs (RMRMs). (B) The analysis of Lasso regression screened thirteen key RMRMs. (C) Cross-validation in the study of Lasso regression for tuning parameter selection. (D) Heatmaps of selected four miRNAs in the training set. (E) The calibration plots confirmed the reliability of the nomogram. (F) A nomogram predicting the 3- and 5-year OS for patients with hepatocellular carcinoma (HCC). (G) Decision curve analysis (DCA) indicates the nomogram offered a significant net benefit over the risk score model and age. [file Image1.tif]

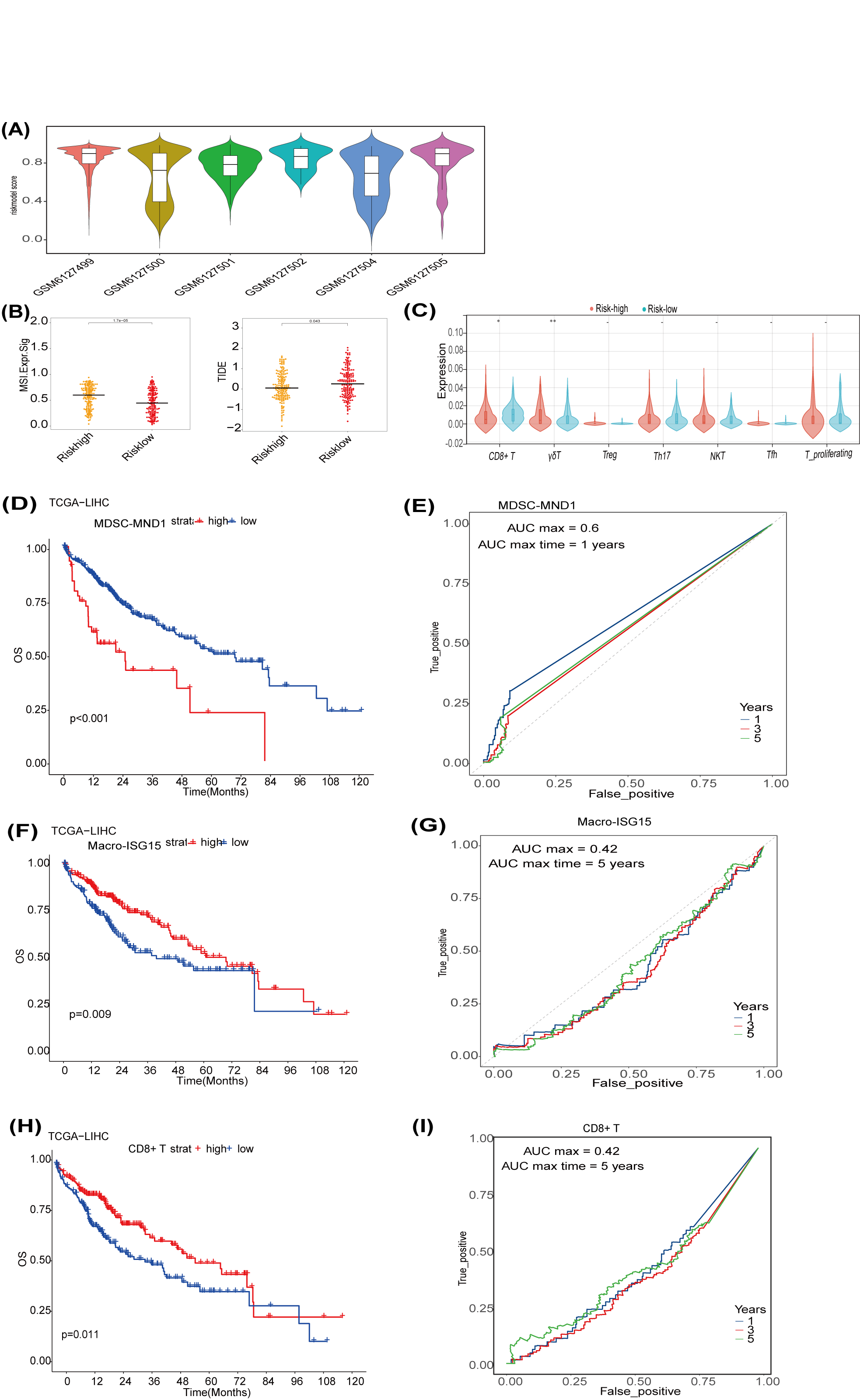

Supplement: Supplementary Figure 2 — Immune characteristics of cell subsets associated with the risk model. (A) The risk model score in each sample in the single-cell RNA sequencing (scRNA-seq) dataset. (B) The correlation between immune infiltration and risk model score in TCGA- hepatocellular carcinoma (HCC). (C) Differential infiltration analysis of T cell subtypes between high- and low-risk model score groups. (D) Kaplan-Meier (KM) survival curves show the association of myeloid-derived suppressor cell (MDSC)- meiotic nuclear divisions 1 (MND1) level with the OS survival of liver hepatocellular carcinoma (LICH) patients. (E) ROC curves show the diagnosis value of MDSC-MND1. (F) KM survival curves show the association of the Mcro-ISG15 level with the OS survival of LICH patients. (G) ROC curves show the diagnosis value of Mcro-ISG15. (H) KM survival curves show the CD8+ T cell level association with the OS survival of LICH patients. (I) ROC curves showing the diagnosis value of CD8+ T cell. [file Image2.tif]

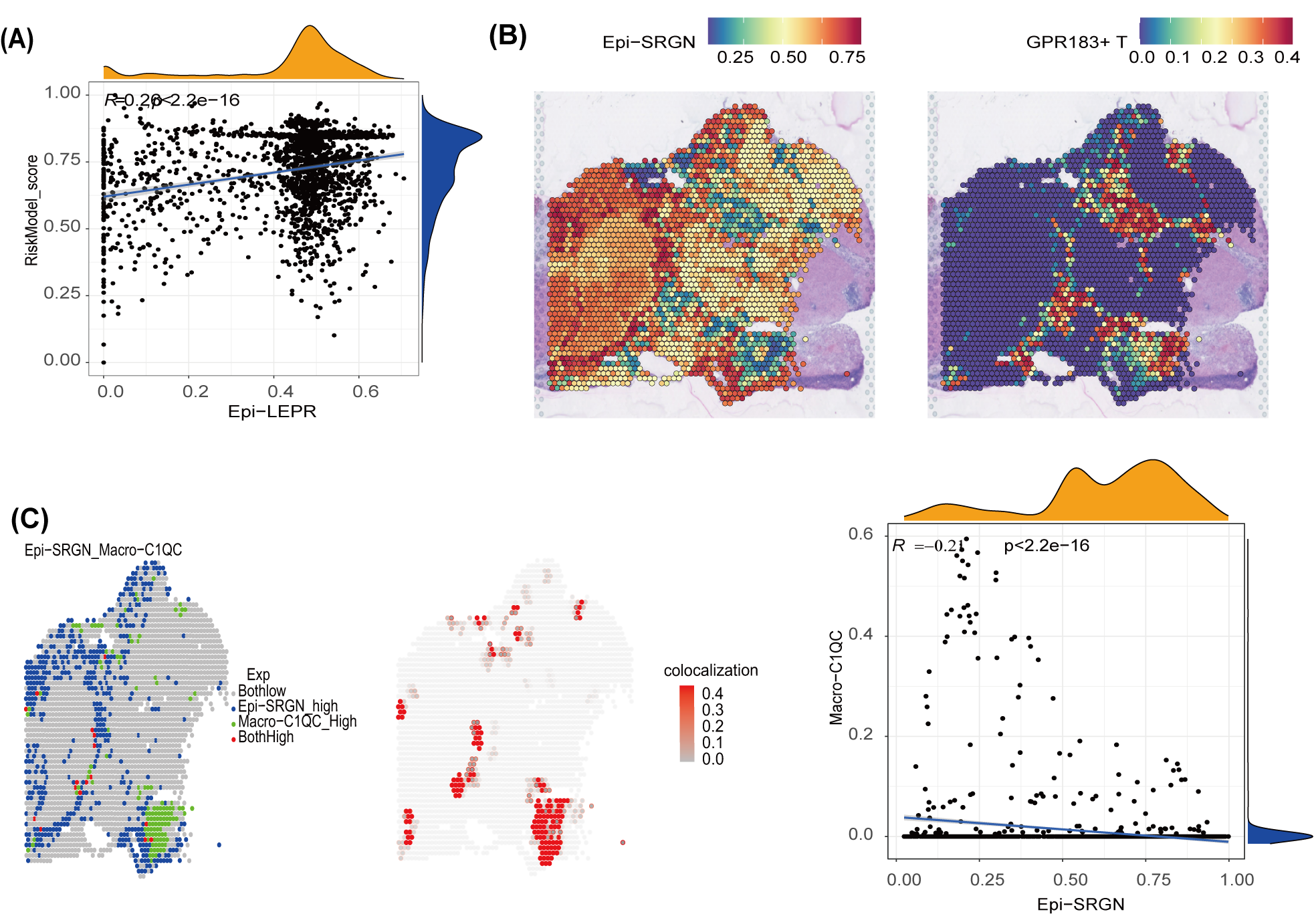

Supplement: Supplementary Figure 3 — Spatial position relationship between risk score, immune cells, and epithelial cells. (A) Spatial correlation between risk score and Epi-LEPR cell. (B) Spatial correlation between Epi-SRGN cell and GPR183+ T cell. (D) Spatial correlation between Macro-C1QC cell and GPR183+ T cell. [file Image3.tif]

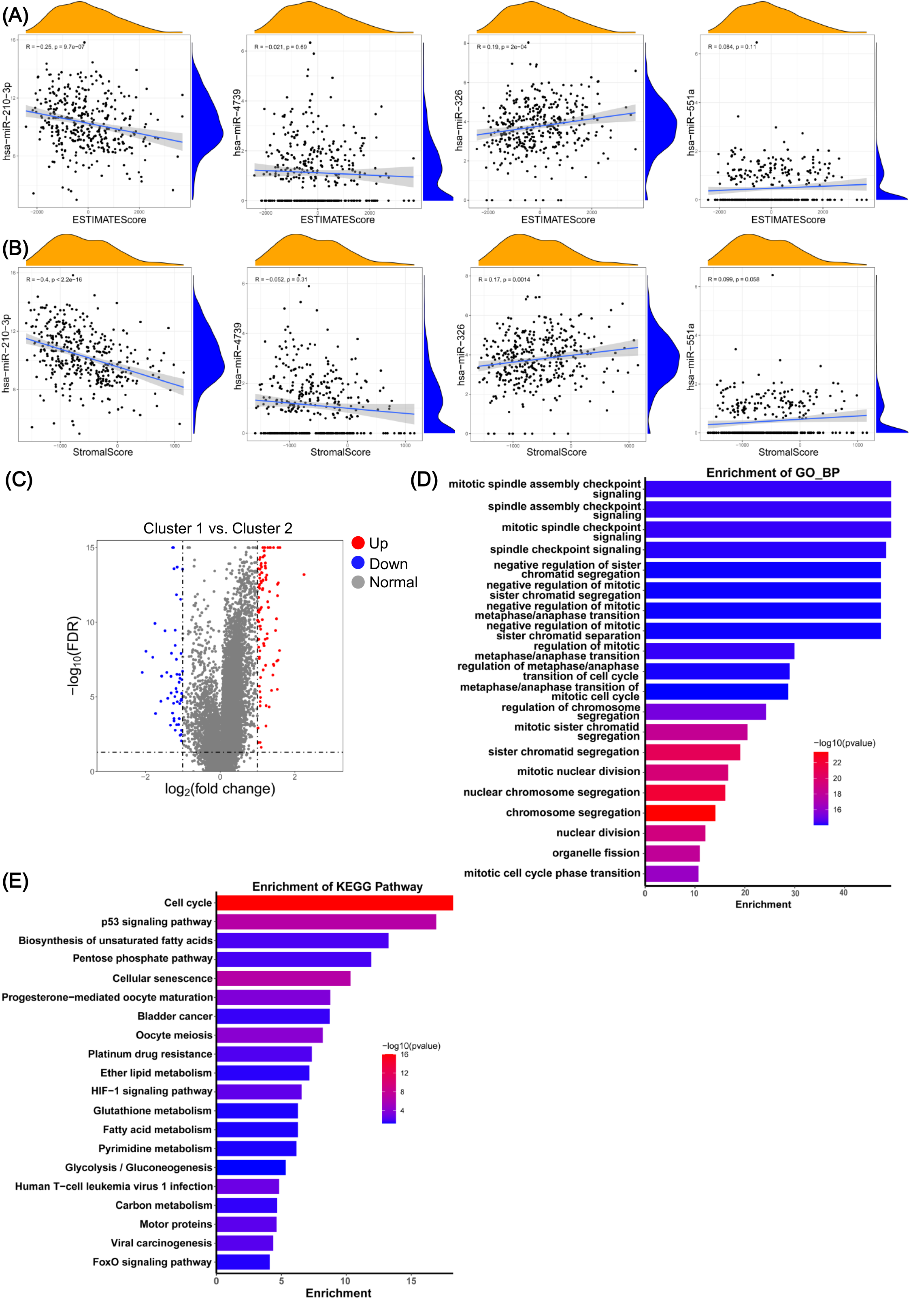

Supplement: Supplementary Figure 4 — Differential analysis for RNA methylation-related miRNA (RMRM)-related clusters. (A, B) The interrelation analysis between the ESTIMATE score and the expression of the four RMRMs (A) and between the stromal score and the expression of the four RMRMs (B). (C) The volcano plot shows the differentially expressed genes (DEGs) between cluster 1 and cluster 2. (D) GO enrichment analysis of upregulated DEGs between cluster 1 and cluster 2. (E) KEGG enrichment analysis of upregulated DEGs between cluster 1 and cluster 2. [file Image4.tif]
